# Supplementary material for: Predictors of injury mortality: findings from a large national cohort in Thailand
Source: BMJ Open. 2014 Jun 5;4(6):e004668. doi: 10.1136/bmjopen-2013-004668 (PMC4054638; doi:10.1136/bmjopen-2013-004668)
Supplement: Author's manuscript [file bmjopen-2013-004668.draft_revisions.pdf]

Predictors of injury mortality: findings from a large national cohort in Thailand

Journal:	*BMJ Open*
Manuscript ID:	bmjopen-2013-004668
Article Type:	Research
Date Submitted by the Author:	11-Dec-2013
Complete List of Authors:	Yiengprugsawan, Dr. Vasoontara; The Australian National University, National Centre for Epidemiology and Population Health<br>Berecki-Gisolf, Janneke; Monash Injury Research Institute,<br>McClure, Roderick; Monash Injury Research Institute,<br>Bain, Christopher; Queensland Institute of Medical Research, Genetics and Population Health Division<br>Seubsman, Sam-ang; Sukhothai Thammathirat Open University,<br>Sleigh, Adrian; The Australian National University, National Centre for Epidemiology and Population Health
**Primary Subject Heading**:	Epidemiology
Secondary Subject Heading:	Epidemiology, Global health, Public health
Keywords:	Epidemiology < THORACIC MEDICINE, Suicide & self-harm < PSYCHIATRY, PUBLIC HEALTH

SCHOLARONE™
Manuscripts

For peer review only

Predictors of injury mortality: findings from a large national cohort in Thailand

Vasoontara Yiengprugsawan[1§], Janneke Berecki-Gisolf[2], Roderick McClure[2], Christopher Bain[3], Sam-ang Seubsman[1,4], Adrian C Sleigh[1] and the Thai Cohort Study Team

[1] National Centre for Epidemiology and Population Health, The Australian National University, Canberra, Australia
[2] Monash Injury Research Institute, Monash University, Melbourne, Australia
[3] Genetics and Population Health Division, Queensland Institute of Medical Research, Brisbane, Queensland, Australia
[4] School of Human Ecology, Sukhothai Thammathirat Open University, Nonthaburi, Thailand

[§] **Correspondence to:** Dr. Vasoontara Yiengprugsawan, The Australian National University, College of Medicine, Biology and Environment, Research School of Population Health, National Centre for Epidemiology and Population Health, Building 62, Mills Rd, Acton 2601, Canberra, Australia Tel: +61 2 6125 2378; Fax: +61 2 6125 0740
Email vasoontara.yieng@anu.edu.au; vasoontara.yieng@gmail.com

For peer review only

Abstract

Objective: to present predictors of injury mortality by types of injury and by pre-existing attributes or other individual exposures identified at baseline.

Design: 5-year prospective longitudinal study

Setting: Contemporary Thailand (2005-2010), a country undergoing epidemiological transition

Participants: Data derived from a research cohort of 87,037 distance-learning students enrolled at Sukhothai Thammathirat Open University (STOU) residing nationwide.

Measures: Cohort members completed a comprehensive baseline mail-out questionnaire in 2005 reporting sociodemographic, behavioural, health, and injury data. These responses were matched with national death records using the Thai Citizen ID number. Age-sex adjusted multinomial logistic regression was used to calculate odds ratios linking exposure variables collected at baseline to injury deaths over the next five years.

Results: Statistically significant predictors of injury mortality were being male (Adjusted Odds Ratio 3.87, 95% Confidence Interval 2.39-6.26), residing in the Southern areas (AOR 1.71, 1.05-2.79), being a current smoker (1.56, 1.03-2.37), history of drink driving (AOR 1.49,1.01-2.20) and ever having been diagnosed for depression (AOR 1.91, 1.00-3.69). Other covariates such as being young, having low social support, and reporting road injury in the past year at baseline had moderately predictive AORs ranging from 1.4 to 1.6 but were not statistically significant.

Conclusions: We complemented routinely collected administration death registration with longitudinal health analyses providing information on individual, social, and health history. This can be used to inform injury prevention policies and strategies suitable for transitional countries with limited resources and changing patterns of mortality.

For peer review only

Article summary:

Article focus

- Injury is a population health burden in low and middle-income Southeast Asian countries.
- We use our large national cohort of Thai adults to investigate risk factors linking to injury mortality including demographic, social, and health attributes provided at the baseline.

Key messages:

- These injury mortality observations add to our previous Thai work on injury morbidity, highlighting the risks connected to depression, male sex, and drink driving.
- The link of the Southern Thai region to non-transport injury deaths probably reflects the political violence which covers the period since 2004.
- Further investigation of deaths by injury types revealed younger age was a strong predictor for transport injury deaths and low social support for non-transport injury deaths.

Strengths and limitations of this study:

- The advantage of our study is its large size, longitudinal design, and comprehensive baseline information. This provides a platform for identification of risks, elimination of confounders, and exploration of causal pathways.
- This study captured the 5-year mortality rate in a generally young adult cohort with relatively few deaths.
- Citizen IDs provided at baseline enable us to monitor patterns of mortality into the future.

For peer review only

Introduction

Injury remains a major public health challenge worldwide; causing one tenth of global mortality with a heavy burden in developing countries.[1, 2] According to the World Health Organization, at least 1.2 million people are killed from road crashes each year and an estimated of 50 million are injured on roads worldwide each year.[3, 4] Violence and non-transport injuries also accounted for more than 1.3 million deaths and many suffered from serious physical and mental consequences.[5, 6]

Most national injury prevention policies have been introduced in high-income countries. Unfortunately, very few low and middle-income countries have been able to develop such policies due to lack of resources and limited availability of quality injury mortality data.[7, 8] In particular, many developing countries still face the challenges of accurately identifying causes of death from routinely collected national civil registration and vital statistics systems; other sources of data such as police reports and hospital records are never comprehensive and lead to underreporting bias if relied upon as a main source of injury mortality data. Reliable cause-of-death data are important because they enable monitoring of the epidemiological occurrence and public health effects at the population level.[9, 10]

Throughout middle-income Southeast Asia, including Thailand, injury continues to be one of the top ten causes of death.[11, 12] In past decades, Thailand has reformed administrative records to improve the coverage and quality of cause-specific mortality data.[13, 14] About 8 years ago, Thailand began to study the ill-defined causes of death by using verbal autopsies and these revealed that besides a high proportion of transport-related deaths, a number of other deaths which were initially recorded as non-specific causes turned out to be suicides, assaults, and drowning.[15-17] These findings shed light on the importance of non-transport injuries in addition to the burden of transport injuries.

This study is based on a large national cohort in Thailand which has investigated health-risk transitions of Thai adults since 2005. The cohort database includes comprehensive information on individual characteristics, social demography, health behaviours and specific diseases, as well as history of injuries. Our previous research based on this cohort has examined risk factors associated with injury morbidity.[18-20] Now successful mortality data linkage through

For peer review only

the Thai Ministry of Interior and Ministry of Public Health allows us to analyse injury-related deaths among the cohort over the first five years. We examine predictors of injury mortality noting links to personal attributes and to exposures reported at baseline.

Methods

Study population and data collection

This analysis is part of the overarching Thai Cohort Study (TCS), an ongoing epidemiological investigation of changing patterns of health risks and outcomes. The resulting pattern has been described as a 'triple threat' – infectious diseases, non-communicable diseases, and injuries.[21, 22] Data derive from a research cohort of 87,037 distance-learning adult students enrolled at Sukhothai Thammathirat Open University (STOU), who resided all over Thailand and completed the baseline comprehensive mail-out health questionnaire in 2005. The cohort participants recapitulated well the distance learning student body at STOU and share similar certain socio-geographic attributes with the general Thai population (mean age was 29 years in 2005, slightly more than half were females, half resided in urban areas).[21, 22] The baseline questionnaire gathered data on a wide range of topics including age, sex, income, marital status, health status, doctored diagnosed diseases, health-risk behaviors including smoking and drinking, social capital, and history of injury.

Mortality data linkage

A powerful feature of this study is that all cohort members have provided their Citizen ID number enabling detection and analysis of deaths into the future. Periodically, the Bangkok TCS team matched death records from the Ministry of Interior using the Thai Citizen ID number. Data released included sex, date of birth, date of death, description in Thai on causes of death. At a later stage the Thai Ministry of Public Health expanded these death records by adding the standard International Classification of Diseases (ICD-10)[23] to identify causes of death.

Up until March 2010, there were a total of 580 deaths among the Thai Cohort Study participants. According to the ICD-10 codes, there were 376 deaths from non-injury causes including ill-defined causes of death. For the purpose of this study, this will not be broken down and will be designated as 'other deaths'. For our injury-focused death analysis, there were 204 deaths from external causes, including 84 deaths from transport accidents. Among the 120

For peer review only

non-transport injury deaths, there were 35 deaths from miscellaneous external causes, 10 deaths from intentional self-harm, 30 deaths from assault, and 45 deaths from 'unspecified events of undetermined intent'.

Exposures and confounders

In this analysis, potential exposures and confounders from the 2005 baseline questionnaire included age categories, sex, marital status, personal household monthly income categories, regions, and lifecourse urbanisation (residence at age 12 years old and at baseline). Health-risk behaviours included smoking, alcohol drinking, and whether ever drink driving in the past year. Other health and social attributes were self-assessed health, chronic metabolic or cardiovascular disorders (e.g. diabetes, hypertension), and ever being diagnosed by doctor for depression. Social capital was captured in three domains: trust (whether people can be trusted), support (from family, friends, colleagues), and interaction (with family, friends, neighbours). As well, injury in the past year was reported at 2005 baseline, including the frequency and location of injuries reported.

Data processing and statistical analysis

Questionnaire responses were digitised by optical scanning and subsequently edited using Thai Scandevet, SQL and SPSS software. For analysis we used Stata version 12. We described the distribution of deaths by demographic, social, and health covariates, by cause of death, and by types of injury (transport and non-transport injuries). In addition, we presented death rates per 10,000 person-years of exposure. We then used age-sex adjusted multinomial logistic regression linking the above covariates to three possible outcomes: alive (reference), deaths from other causes, and deaths from injury (study outcome). The final analytical model mutually adjusted for all covariates and reported Adjusted Odds Ratios (AOR) and 95% Confidence Intervals.

Results

Among Thai cohort members at baseline in 2005 (Table 1), about half were less than 30 years old, slightly more than half were female, about 40% reported monthly income of less than 7000 Baht per month (175 US$ in 2005), 30% lived in Bangkok, and about half were urban residents. Injury deaths were more likely to affect males (73.7%, 15 vs 23 per 10000 person-

For peer review only

years for transport vs non-transport injuries). Also notable, injury deaths were disproportionately frequent in the Southern region (20.6%, 12 vs 25 per 10000 person-years for transport vs non-transport injuries).

For social and health attributes (Table 2), a history of ever drink driving in the past year was more common among injury deaths (42.4% compared to 26.5% for 'other deaths or 25.4% for alive) and notably higher for transport than non-transport injuries (52.9% vs 35.0%). Cohort members who died from non-injury related causes were twice as likely to have reported at baseline poor self-assessed health and three times as likely to have reported metabolic and cardiovascular chronic conditions. Cohort members who died from injury reported higher rates of ever having been doctor-diagnosed depression (6.9% compared to 3.4% among non-deaths). As well, a history of depression was much more frequent for those who died from non-transport injuries than for transport injuries (34 vs 13 per 10000 person-years). At the baseline 2005, about 20% overall cohort members reported injury at least once in the past year compared to 33.3% of cohort participants who died from transport injury.

In addition to analysing by injury types, we also tabulated the death rates according to the International Classification of Diseases (Table 3). Within transport injury mortality, rates per 10000 person-years for motorcycle riders and car occupants were 1.6 and 1.7, respectively. There were also 4.5 per 10000 person-years who died in unspecified motor vehicles. Among non-transport injury deaths, the rate per 10000 person-years of assault by firearm discharge was 2.5 with an additional 1.5 deaths from firearm discharge with undetermined intent. Deaths from drowning and submersion were 1.3 per 10000 person-years. Intentional self-harm deaths included self-poisoning and hanging-strangulation-suffocation with death rates of 0.3 and 0.8 per 10000 person-years, respectively.

To examine predictors of injury deaths (Table 4), we used multinomial logistic regression with three outcome categories: alive (reference), non-injury deaths, and injury deaths (study outcome). Highlighted in bold were results that were statistically significant at $p<0.05$. In the first column of odds ratios, the results are adjusted for age and sex; in the second column the odds ratios are adjusted for all covariates. All odds ratios compare the odds of injury death to the odds of staying alive.

We first calculate age-sex adjusted odds ratios for each potential exposure and for covariates. Among injury deaths in the age-sex adjusted model, being younger than 30 years,

For peer review only

males, residing in the South, current smoker, history of drink driving in the past year, ever having been diagnosed for depression, having low social support, reported history of injuries in the past year at baseline, and reported road injury in 2005.

We then proceed to multivariate analysis (Table 4). After mutually adjusting for all tabulated covariates, statistically significant predictors of injury mortality were being male (Adjusted Odds Ratio 3.87, 95% CI 2.39-6.26), residing in the Southern areas (AOR 1.71, 1.05-2.79), being a current smoker (1.56, 1.03-2.37), history of drink driving (AOR 1.49,1.01-2.20) and ever having been diagnosed for depression (AOR 1.91, 1.00-3.69). Other covariates such as being young, having low social support, and reporting road injury in the past year at baseline had predictive AORs ranging from 1.4 to 1.6, but these substantive estimates were not statistically significant.

In marked contrast to the injury deaths, cohort members who died from other causes had different sets of statistically significant risks at 2005 baseline which included older age, residing in Bangkok, reporting poor self-assessed health, and having metabolic or cardiovascular chronic conditions (data not shown). The only risk factor that non-injury deaths have in common with injury deaths was being a current smoker.

Discussion

This study is embedded in an overarching investigation of health-risk transition in Thailand, where like other middle-income Southeast Asian countries; injury has been and still is a major population health burden. We make use of our large cohort of adults to investigate risk factors associated with injury mortality, linking deaths over five years (2005-2010) to individual characteristics and social and health attributes recorded at baseline (2005). The results revealed that injuries constituted almost one third of all deaths in the cohort, and 40% of those were from transport and 60% were non-transport injuries. Connecting to baseline information provided five years earlier, we identified epidemiologically and statistically significant predictors of injury mortality. These predictors included certain geo-demographic characteristics (male sex, Southern residents), health risk behaviours (smoking and history of drink driving), and adverse health diagnoses (depression).

For peer review only

Our injury mortality findings provide further information adding to our previous knowledge on risk factors associated with injury morbidity in the cohort, especially being male, having a history of depression, and drink driving.[18-20] Non-transport injury deaths presented here reflected the current political situation with violence in the Southern part of Thailand since 2004.[24, 25] For covariates with substantial (but not statistically significant) point estimates of overall injury death (young age, low social support, history of road injury), AORs ranged from 1.4-1.6. Further investigation into types of injury (data not shown) revealed that, younger age was a strong predictor for transport injury deaths (AOR 4.12, 95% 1.03-16.5) and low social support for non-transport injury deaths (AOR 1.64, 95% 1.04-2.59).

Our results support other published international information on risk factors related to injury mortality, showing young males and drink drivers at high risk of road crash death.[26, 27] A systematic review on alcohol consumption and collision risk concluded that there is no safe level of drinking and even less than two drinks per occasion can almost double the odds of most types of injury.[26] Our study also found the protective role of social capital, again supporting previous research;[28-30] however in Thailand we found social support to be predictor of non-transport injury mortality but not for overall injury deaths. Despite the relatively small number of deaths in our study, we found that low social support was particularly associated with intentional self-harm and assault but these associations were not statistically significant.

The advantage of our study is its large participation by Thai adults who completed a comprehensive 20-page baseline questionnaire in 2005, reporting on a wide array of social and health characteristics. This provided a platform for investigation of causal pathways and elimination of many confounders. While our cohort members share similar distribution of sex, modest income, and geographical residence with the general Thai population, we noted that they had all completed high school level which facilitated their ability to respond to our detailed questionnaire. We also note that this study captured the 5-year mortality rate after the baseline questionnaire in 2005 and the number of deaths was relatively small. The citizen IDs provided by cohort members at the baseline can be used to monitor patterns of mortality into the future and relating it to baseline and follow-up exposures.

Our research contributes to limited longitudinal evidence linking risk factors to injury death and is one of the first studies in middle-income Southeast Asia. We have achieved our aim of identifying vulnerable population subgroups at risk of injury deaths. As well, we

For peer review only

complemented routinely collected administration death registration with a longitudinal health assessment providing information on individual, social, and health history factors. This information is invaluable in yielding insight into certain risk traits such as being a young male, reporting drink driving, and having a past medical diagnosis of depression, which could inform injury prevention policies and strategies suitable for transitional countries with limited resources and changing patterns of mortality.

For peer review only

Contributors

VY conceptualized, analysed and drafted the manuscript. JB-G, RM, CB provided inputs to improve the draft. SS and AS designed and executed the Thai Cohort Study. AS provided comments and guidance on various revisions. All authors read and approved the final manuscript.

Author footnote

The Thai Cohort Study Team

Thailand: Jaruwan Chokhanapitak, Suttanit Hounthasarn, Suwanee Khamman, Daoruang Pandee, Suttinan Pangsap, Tippawan Prapamontol, Janya Puengson, Sam-ang Seubsman, Boonchai Somboonsook, Nintita Sripaiboonkij, Pathumvadee Somsamai, Duangkae Vilainerun, Wanee Wimonwattanaphan, Tewarit Somkotra, Benjawan Tawatsupa, Wimalin Rimpeekool.

Australia: Chris Bain, Emily Banks, Cathy Banwell, Bruce Caldwell, Gordon Carmichael, Tarie Dellora, Jane Dixon, Sharon Friel, David Harley, Matthew Kelly, Tord Kjellstrom, Lynette Lim, Anthony McMichael, Tanya Mark, Penny Haora, Adrian Sleigh, Lyndall Strazdins, Susan Jordan, Roderick McClure, Janneke Berecki-Gisolf, Vasoontara Yiengprugsawan.

Acknowledgments

We thank the staff at Sukhothai Thammathirat Open University (STOU) who assisted with student contact and the STOU students who are participating in the cohort study. We also thank Dr Bandit Thinkamrop and his team from Khon Kaen University for support on initial data processing. As well we would like thank the Thai Ministry of Interior and Ministry of Public Health for mortality investigation and cause of death data linkage.

Funding

This study was supported by the International Collaborative Research Grants Scheme with joint grants from the Wellcome Trust UK (GR071587MA) and the Australian National Health and Medical Research Council (268055), and as a global health grant from the NHMRC (585426).

Ethical approval

Informed written consent was obtained from all participants. All students were advised that they could withdraw, or not participate, without any effect on their academic progress. They were assured that their information was confidential and their identity would never be revealed. The questionnaires never sought sensitive personal information and no biological samples were taken. Ethics approval was obtained from Sukhothai Thammathirat Open University Research and Development Institute (protocol 0522/10) and the Australian National University Human Research Ethics Committee (protocols 2004/344 and 2009/570).

Competing interests

None declared.

Data sharing statement

Data are available through a data access agreement which includes guarantees regarding ethical conduct and scientific quality of any proposed analyses and publications. Anyone wanting access should contact Professor Adrian Sleigh or Associate Professor Sam-ang Seubsman (Thai Cohort Study Principal Investigators).

For peer review only

Table 1 Distribution of mortality by baseline social and geo-demographic attributes, Thai Cohort Study

Cohort attributes	Vital status by attributes %					Incidence per 10000 person-years	
	Alive (86457)	Other deaths (376)	Injury deaths (204)	Injury deaths (204)		Transport (84)	Non-transport (120)
				Transport (84)	Non-transport (120)		
**Age groups in years:**							
18-29	31.4	27.7	27.9	20.2	25.7	6	15
30-39	53.6	29.5	59.3	69.1	51.4	12	14
40-49	12.5	25.8	9.3	6.0	17.1	5	13
≥50	2.4	17.0	3.4	4.8	5.7	19	14
**Sex:**							
Males	45.2	65.6	73.7	69.4	76.7	15	23
**Marital status:**							
Married	38.8	50.1	35.0	30.9	37.7	8	14
Not married	56.8	41.9	60.1	64.3	57.1	11	14
Divorced/widowed	4.4	8.0	4.9	4.8	5.0	11	16
**Personal monthly income:**							
≤3000 Baht	11.0	14.9	14.6	11.9	16.5	10	20
3001-7000	30.9	22.4	33.2	39.3	28.7	13	13
7001-10000	23.3	16.6	23.6	26.2	21.7	11	13
10001-20000	24.2	28.7	20.6	16.7	23.5	7	13
>20000	10.5	17.4	8.0	6.0	9.6	6	12
**Regions:**							
Central/East	30.7	30.7	24.9	23.8	24.2	8	11
Bangkok	17.2	18.7	10.7	9.4	11.7	5	9
North	18.2	22.7	21.0	23.5	19.2	13	15
Northeast	20.9	21.6	23.4	25.9	21.7	12	14
South	13.0	12.3	20.6	16.7	23.3	12	25
**Lifecourse residence:**							
Rural-rural	43.3	42.3	46.6	38.1	52.5	8	17
Rural-urban	31.5	25.0	16.7	28.6	30.8	9	13
Urban-rural	4.2	6.9	4.4	7.1	2.5	16	8
Urban-urban	19.7	22.3	16.7	22.6	12.5	11	9

For peer review only

Table 2 Mortality by baseline health-risk behaviours and states, social attributes, and history of injury, Thai Cohort Study

Social and health attributes	Vital status by attributes %					Incidence per 10000 person-years	
	Alive (86457)	Other deaths (376)	Injury deaths (204)	Injury deaths (204)		Transport (84)	Non-transport (120)
				Transport (84)	Non-transport (120)		
**Health-risk attributes**							
**Smoking:**							
Never	72.3	51.1	57.5	66.3	51.3	9	10
Current	10.0	19.8	24.0	21.7	25.6	21	35
Former	15.8	26.1	15.5	9.6	19.7	6	17
**Alcohol drinking:**							
Never	26.5	22.9	19.8	21.4	18.6	8	10
Occasional	59.8	49.5	60.9	61.9	60.2	10	14
Regular	4.8	9.7	7.9	9.5	6.8	19	19
Stop	8.9	18.0	11.4	7.1	14.4	8	22
**Ever drink drive in past year:**							
Yes	25.4	26.5	42.4	52.9	35.0	20	19
Not usually drive	8.8	8.2	8.8	9.4	8.3	10	13
**Health and social attributes**							
**Self-assessed health:**							
Poor or very poor	4.6	8.8	3.9	2.4	5.0	7	16
**Chronic conditions:**							
Yes	12.5	29.3	10.8	13.1	9.2	10	13
**Doctor diagnosed depression:**							
Yes	3.4	5.9	6.9	4.8	8.3	13	34
**Social capital:**							
Low trust	38.2	36.9	34.5	35.4	33.9	9	12
Low support	25.5	33.2	20.1	22.6	18.3	9	20
Low interaction	23.3	25.5	28.9	22.6	33.3	9	10
**Injury reported in 2005**							
**Number of injury:**							
At least once	20.2	29.5	27.9	33.3	24.2	16	16
**Location of injury:**							
Home	5.3	7.1	5.4	5.9	5.0	10	14
Road	5.9	4.8	11.7	16.5	8.3	25	20
Work	3.9	6.4	5.4	9.5	2.5	11	13

For peer review only

Table 3 Injury mortality by International Classification of Diseases (ICD-10), Thai Cohort Study

Types of injury deaths	Number of deaths	Rate per 10000 person-years
**Transport injuries**		
V01-V09 Pedestrian	2	0.2
V20-V29 Motorcycle rider	14	1.6
V40-V49 Car occupant	15	1.7
V50-59 Occupant of pick-up truck or van	3	1.0
V80-V89 Other land transport accident	9	0.1
V89.2 Person injured in unspecified motor vehicle	39	4.5
V90-V94 Water transport	1	0.1
V95-V97 Air and space transport	1	0.1
**Non-transport injuries**		
W00-W19 Falls	2	0.3
W65-W74 Drowning and submersion	10	1.3
W75-W84 Other threats to breathing	1	0.1
W87 Exposure to electric current	2	0.3
X00-X09 Exposure to smoke, fire and flames	2	0.3
X33 Victim of lighting	1	0.1
X38 Victim of flood	1	0.1
X58-X59 Exposure to other unspecified factors	16	2.1
X60-X84 Intentional self-harm		
X65 Intentional self-poisoning	3	0.3
X70 Intentional self-harm by hanging, strangulation and suffocation	7	0.8
X85-Y09 Assault		
X95 Assault by unspecified firearm discharge	22	2.5
X99 Assault by sharp object	3	0.3
Y99 Assault by other unspecified means	5	0.6
Y10-Y34 Event of undetermined intent		
Y18 Poisoning by and exposure to pesticides	1	0.1
Y20 Hanging, strangulation and suffocation	3	0.3
Y22-Y24 Firearm discharge, undetermined intent	13	1.5
Y25 Contact with explosive material	1	0.1
Y28-Y29 Contact with sharp of blunt object	4	0.5
Y34 Unspecified event, undetermined intent	23	2.6

For peer review only

Table 4 Age-sex adjusted and multivariate predictors of injury mortality, Thai Cohort Study

2005 baseline covariates	Multinomial* Adjusted Odds Ratios [95% CI]			
	Age-sex adjusted		Multivariate**	
	Alive	Injury death	Alive	Injury death
**Demo-geographic covariates**				
**Age groups in years:** 20-29		**1.45 [1.06-1.99]**		1.51 [0.99-2.32]
30-39	*Ref*		*Ref*	
40-49		0.76 [0.45-1.28]		0.99 [0.54-1.83]
≥50		1.29 [0.59-2.84]		1.65 [0.61-4.43]
**Sex** *(female)*:	*Ref*		*Ref*	
Male		**3.69 [2.69-5.07]**		**3.87[2.39-6.26]**
**Marital status** *(married)*:	*Ref*		*Ref*	
Not married		1.09 [0.77-1.56]		1.11 [0.73-1.69]
Divorced/widowed		1.55 [0.80-3.02]		1.47 [0.69-3.14]
**Personal monthly income:**				
≤3000Baht		1.24 [0.80-1.93]		1.10 [0.48-1.53]
3001-7000		1.25 [0.89-1.75]		1.15 [0.78-1.69]
7001-20000		0.81 [0.46-1.43]		0.83 [0.43-1.61]
>20000	*Ref*		*Ref*	
**Regions** (*Central/East*):	*Ref*		*Ref*	
Bangkok		0.85 [0.51-1.41]		0.86 [0.48-1.53]
North		1.38 [0.92-2.08]		1.27 [0.80-2.01]
Northeast		1.29 [0.87-1.93]		0.98 [0.61-1.56]
South		**1.96 [1.30-2.97]**		**1.71 [1.05-2.79]**
**Lifecourse residence** *(rural-rural)*:	*Ref*		*Ref*	
Urban-rural		0.91 [0.66-1.27]		0.97 [0.65-1.44]
Rural-urban		1.03 [0.52-2.04]		1.32 [0.73-1.44]
Urban-urban		0.87 [0.59-1.29]		1.12 [0.71-1.77]
**Health and social covariates**				
**Smoking** *(never)*:	*Ref*		*Ref*	
Current		**1.70 [1.07-2.05]**		**1.56 [1.03-2.37]**
Former		0.77 [0.45-1.32]		0.74 [0.47-1.18]
**Alcohol drinking** *(never)*:	*Ref*		*Ref*	
Occasional		0.87 [0.60-1.27]		0.40 [0.12-1.30]
Regular		1.06 [0.58-1.95]		0.38 [0.10-1.40]
Stop		1.08 [0.64-1.84]		0.63 [0.18-2.15]
**Drink drive past year** *(never)*:	*Ref*		*Ref*	
Yes		**1.50 [1.07-2.12]**		**1.49 [1.01-2.20]**
**Self-assessed health** *(good)*:	*Ref*		*Ref*	
Poor or very poor		1.09 [0.58-2.07]		0.75 [0.32-1.71]
**Depression** *(no)*:	*Ref*		*Ref*	
Yes		**2.15 [1.25-3.71]**		**1.91 [1.00-3.69]**
**Chronic illness** *(no)*:	*Ref*		*Ref*	
Yes		0.80 [0.50-1.2]		0.84 [0.50-1.44]
**Social capital:**				
Social trust (*ref: no*)		084 [0.60-1.19]		0.90 [0.76-1.07]
Social support (*ref*: no)		1.29 [0.58-2.84]		1.37 [0.96-1.96]
Social interaction (*ref*: no)		0.86 [0.64-1.15]		0.77 [0.50-1.20]
**Injury reported in 2005**				
**Injuries in the past year** *(no)*:	*Ref*		*Ref*	
At least once		**1.41 [1.04-1.92]**		1.12 [0.70-1.82]
**Location of injury:**				
Home (*ref: no*)		1.13 [0.62-2.08]		1.19 [0.56-2.55]
Road (*ref: no*)		**1.81 [1.17-2.79]**		1.58 [0.87-2.84]
Work (*ref: no*)		1.22 [0.66-2.25]		1.15 [0.54-2.44]

*multinomial logistic regression compares the odds of injury deaths to the odds of remaining alive by predictor covariate category values, after adjusting for age-sex or other covariates.

**mutually adjusted for all predictor covariates presented in this table

For peer review only

References

1. Stevenson M. Measuring the global burden of road traffic injury: implications for low-income and middle-income countries. Inj Prev. 2009; 15:1-2.
2. Lim SS, Vos T, Flaxman AD, Danaei G, Shibuya K, Adair-Rohani H, et al. A comparative risk assessment of burden of disease and injury attributable to 67 risk factors and risk factor clusters in 21 regions, 1990-2010: a systematic analysis for the Global Burden of Disease Study 2010. Lancet. 2012; 380:2224-60.
3. WHO. Global status report on road safety 2013: supporting a decade of action. Geneva: World Health Organization; 2013.
4. WHO. World report on road traffic injury prevention. Geneva: World Health Organization; 2004.
5. Chandran A, Hyder AA, Peek-Asa C. The global burden of unintentional injuries and an agenda for progress. Epidemiol Rev. 2010; 32:110-20.
6. WHO. Global status report on violence prevention. Geneva: World Health Organization; 2014.
7. Bhalla K, Harrison J, Abraham J, Borse NN, Lyons R, Boufous S, et al. Data sources for improving estimates of the global burden of injuries: call for contributors. PLoS Med. 2009; 6:e1.
8. Bhalla K, Harrison JE, Fingerhut LA, Shahraz S, Abraham J, Yeh PH, et al. The global injury mortality data collection of the Global Burden of Disease Injury Expert Group: a publicly accessible research tool. Int J Inj Contr Saf Promot. 2011; 18:249-53.
9. Baker SP, Li G. Epidemiologic approaches to injury and violence. Epidemiol Rev. 2011; 34:1-3.
10. Mathers CD, Fat DM, Inoue M, Rao C, Lopez AD. Counting the dead and what they died from: an assessment of the global status of cause of death data. Bulletin of the World Health Organization [NLM - MEDLINE]. 2005; 83:171.
11. Ditsuwan V, Veerman LJ, Barendregt JJ, Bertram M, Vos T. The national burden of road traffic injuries in Thailand. Popul Health Metr. 2011; 9:2.
12. Chadbunchachai W, Suphanchaimaj W, Settasatien A, Jinwong T. Road traffic injuries in Thailand: current situation. J Med Assoc Thai. 2012; 95 Suppl 7:S274-81.
13. Choprapawon C, Porapakkham Y, Sablon O, Panjajaru R, Jhantharatat B. Thailand's national death registration reform: verifying the causes of death between July 1997 and December 1999. Asia Pac J Public Health. 2005; 17:110-6.
14. Tangcharoensathien V, Faramnuayphol P, Teokul W, Bundhamcharoen K, Wibulpholprasert S. A critical assessment of mortality statistics in Thailand: potential for improvements. Bull World Health Organ. 2006; 84:233-8.
15. Rao C, Porapakkham Y, Pattaraarchachai J, Polprasert W, Swampunyalert N, Lopez AD. Verifying causes of death in Thailand: rationale and methods for empirical investigation. Popul Health Metr. 2010; 8:11.
16. Porapakkham Y, Rao C, Pattaraarchachai J, Polprasert W, Vos T, Adair T, et al. Estimated causes of death in Thailand, 2005: implications for health policy. Popul Health Metr. 2010; 8:14.
17. Pattaraarchachai J, Rao C, Polprasert W, Porapakkham Y, Pao-In W, Singwerathum N, et al. Cause-specific mortality patterns among hospital deaths in Thailand: validating routine death certification. Popul Health Metr. 2010; 8:12.
18. Yiengprugsawan V, Stephan K, McClure R, Kelly M, Seubsman S, Bain C, et al. Risk factors for injury in a national cohort of 87,134 Thai adults. Public Health. 2012; 126:33-9.
19. Stephan K, Kelly M, McClure R, Seubsman SA, Yiengprugsawan V, Bain C, et al. Distribution of transport injury and related risk behaviours in a large national cohort of Thai adults. Accid Anal Prev. 2011; 43:1062-7.
20. Tawatsupa B, Yiengprugsawan V, Kjellstrom T, Berecki-Gisolf J, Seubsman SA, Sleigh A. Association between heat stress and occupational injury among Thai workers: findings of the Thai Cohort Study. Ind Health. 2013; 51:34-46.
21. Sleigh AC, Seubsman SA, Bain C. Cohort profile: The Thai Cohort of 87,134 Open University students. Int J Epidemiol. 2008; 37:266-72.
22. Seubsman S, Yiengprugsawan V, Sleigh A. A large national Thai Cohort Study of the Health-Risk Transition based on Sukhothai Thammathirat Open University students. ASEAN Journal of Open and Distance Learning. 2012; 4:58-69.
23. WHO. International Statistical Classification of Diseases and Related Health Problems, 10th Revision. http://apps.who.int/classifications/icd10/browse/2010/en: World Health Organization.; 2011.
24. Bonura C. Indeterminate geographies of political violence in Southern Thailand. Alternatives. 2008; 33:383-412.
25. McCargo D. Rethinking Thailand's southern violence - Introduction. Crit Asian Stud. 2006; 38:3-10.

For peer review only - http://bmjopen.bmj.com/site/about/guidelines.xhtml

26. Taylor B, Irving HM, Kanteres F, Room R, Borges G, Cherpitel C, et al. The more you drink, the harder you fall: a systematic review and meta-analysis of how acute alcohol consumption and injury or collision risk increase together. Drug Alcohol Depend. 2010; 110:108-16.
27. Connor J, Norton R, Ameratunga S, Jackson R. The contribution of alcohol to serious car crash injuries. Epidemiology. 2004; 15:337-44.
28. Aida J, Kondo K, Hirai H, Subramanian SV, Murata C, Kondo N, et al. Assessing the association between all-cause mortality and multiple aspects of individual social capital among the older Japanese. BMC Public Health. 2011; 11:499.
29. Hyyppa MT, Maki J, Impivaara O, Aromaa A. Individual-level measures of social capital as predictors of all-cause and cardiovascular mortality: a population-based prospective study of men and women in Finland. Eur J Epidemiol. 2007; 22:589-97.
30. Muennig P, Cohen AK, Palmer A, Zhu W. The relationship between five different measures of structural social capital, medical examination outcomes, and mortality. Soc Sci Med. 2013; 85:18-26.

For peer review only

BMJ Open

Predictors of injury mortality: findings from a large national cohort in Thailand

Journal:	*BMJ Open*
Manuscript ID:	bmjopen-2013-004668.R1
Article Type:	Research
Date Submitted by the Author:	15-Apr-2014
Complete List of Authors:	Yiengprugsawan, Dr. Vasoontara; The Australian National University, National Centre for Epidemiology and Population Health<br>Berecki-Gisolf, Janneke; Monash Injury Research Institute,<br>McClure, Roderick; Monash Injury Research Institute,<br>Bain, Christopher; Queensland Institute of Medical Research, Genetics and Population Health Division<br>Seubsman, Sam-ang; Sukhothai Thammathirat Open University,<br>Sleigh, Adrian; The Australian National University, National Centre for Epidemiology and Population Health
**Primary Subject Heading**:	Epidemiology
Secondary Subject Heading:	Epidemiology, Global health, Public health
Keywords:	Epidemiology < THORACIC MEDICINE, Suicide & self-harm < PSYCHIATRY, PUBLIC HEALTH

SCHOLARONE™
Manuscripts

For peer review only

Predictors of injury mortality: findings from a large national cohort in Thailand

Vasoontara Yiengprugsawan[1§], Janneke Berecki-Gisolf[2], Roderick McClure[2], Christopher Bain[3], Sam-ang Seubsman[1,4], Adrian C Sleigh[1]

[1] National Centre for Epidemiology and Population Health, The Australian National University, Canberra, Australia
[2] Monash Injury Research Institute, Monash University, Melbourne, Australia
[3] Genetics and Population Health Division, Queensland Institute of Medical Research, Brisbane, Queensland, Australia
[4] School of Human Ecology, Sukhothai Thammathirat Open University, Nonthaburi, Thailand

[§] **Correspondence to:** Dr. Vasoontara Yiengprugsawan, The Australian National University, College of Medicine, Biology and Environment, Research School of Population Health, National Centre for Epidemiology and Population Health, Building 62, Mills Rd, Acton 2601, Canberra, Australia Tel: +61 2 6125 2378; Fax: +61 2 6125 0740
Email vasoontara.yieng@anu.edu.au; vasoontara.yieng@gmail.com

For peer review only

Predictors of injury mortality: findings from a large national cohort in Thailand

Abstract

Objective: to present predictors of injury mortality by types of injury and by pre-existing attributes or other individual exposures identified at baseline.

Design: 5-year prospective longitudinal study

Setting: Contemporary Thailand (2005-2010), a country undergoing epidemiological transition

Participants: Data derived from a research cohort of 87,037 distance-learning students enrolled at Sukhothai Thammathirat Open University (STOU) residing nationwide.

Measures: Cohort members completed a comprehensive baseline mail-out questionnaire in 2005 reporting geo-demographic, behavioural, health, and injury data. These responses were matched with national death records using the Thai Citizen ID number. Age-sex adjusted multinomial logistic regression was used to calculate odds ratios linking exposure variables collected at baseline to injury deaths over the next five years.

Results: Statistically significant predictors of injury mortality were being male (Adjusted Odds Ratio 3.87, 95% Confidence Interval 2.39-6.26), residing in the Southern areas (AOR 1.71, 1.05-2.79), being a current smoker (1.56, 1.03-2.37), history of drink driving (AOR 1.49,1.01-2.20) and ever having been diagnosed for depression (AOR 1.91, 1.00-3.69). Other covariates such as being young, having low social support, and reporting road injury in the past year at baseline had moderately predictive AORs ranging from 1.4 to 1.6 but were not statistically significant.

Conclusions: We complemented national death registration with longitudinal data on individual, social, and health attributes. This information is invaluable in yielding insight into certain risk traits such as being a young male, history of drink driving, and history of depression. Such information could be used to inform injury prevention policies and strategies.

Article summary:

Article focus

- Injury is a population health burden in transitional low and middle-income Southeast Asia.
- We investigated a large national cohort of Thai adults for predictors of injury mortality including geo-demographic, social, and health attributes recorded at baseline.

Key messages:

- Injuries constituted almost one third of all deaths in the cohort, and some 40% of those were from transport and nearly 60% were non-transport injuries.
- These injury mortality observations add to our previous Thai work on injury morbidity, highlighting the overall risks, especially depression, male sex, and drink driving.
- The link of the Southern Thai region to non-transport injury deaths most likely reflects the political violence which covers the period since 2004.

Strengths and limitations of this study:

- The advantage of our study is its large size, longitudinal design, and comprehensive baseline information. This provides a platform for identification of risks, elimination of confounders, and exploration of causal pathways.
- This study captured the 5-year mortality rate in a generally young adult cohort. Thus there were relatively few deaths.
- Citizen IDs provided at baseline will enable us to monitor patterns of cohort mortality into the future.

For peer review only

Introduction

Injury remains a major public health challenge worldwide; causing one tenth of global mortality with a heavy burden in developing countries.[1, 2] According to the World Health Organization, at least 1.2 million people are killed from road crashes and an estimated 50 million are injured on roads worldwide each year.[3, 4] Violence and non-transport injuries also accounted for more than 1.3 million deaths and many suffered from serious physical and mental consequences.[5, 6]

Most national injury prevention policies have been introduced in high-income countries. Unfortunately, very few low and middle-income countries have been able to develop such policies due to lack of resources and limited availability of quality injury mortality data.[7, 8] In particular, many developing countries still face the challenges of accurately identifying causes of death from routinely collected national civil registration and vital statistics systems while other sources of data such as police reports and hospital records are never comprehensive and lead to underreporting bias if relied upon as a main source of injury mortality data. Reliable cause-of-death data are important because they enable monitoring of the epidemiological occurrence and public health effects at the population level.[9, 10]

Throughout middle-income Southeast Asia, including Thailand, injury continues to be one of the top ten causes of death.[11, 12] In past decades, Thailand has reformed administrative records to improve the coverage and quality of cause-specific mortality data.[13, 14] Eight years ago, Thailand began to study the ill-defined causes of death by using verbal autopsies and these revealed that besides a high proportion of transport-related deaths, a number of other deaths which were initially recorded as non-specific causes turned out to be suicides, assaults, and drowning.[15-17] These findings shed light on the importance of non-transport injuries in addition to the burden of transport injuries.

This study is based on a large national cohort in Thailand which has been followed to investigate health-risk transitions of Thai adults since 2005. The cohort database includes comprehensive information on individual characteristics, social demography, health behaviours and specific diseases, as well as history of injuries. Our previous research based on this cohort has examined risk factors associated with injury morbidity.[18-20] Now successful mortality data linkage

For peer review only

through the Thai Ministry of Interior and Ministry of Public Health allows us to analyse injury-related deaths among the cohort over the first five years (2005-10).

Informed by our earlier research on injury morbidity, and by related published information, this study has investigated injury in more depth using mortality as the outcome. Our study linked cohort outcomes (survival, non-injury death, injury death) to an array of relevant exposures recorded at baseline including geo-demographic attributes, social covariates, health and psychological states, and health-risk behaviours. This analysis is both prospective and cohort-based and fills an important gap regarding our knowledge of injury risks as an emerging public health problem in a middle-income Asian country going through the health-risk transition.

Methods

Study population and data collection

This analysis is part of the overarching Thai Cohort Study (TCS), an ongoing epidemiological investigation of changing patterns of health risks and outcomes. Data derive from a research cohort of 87,037 distance-learning adult students enrolled at Sukhothai Thammathirat Open University (STOU), who resided all over Thailand and completed the baseline comprehensive mail-out health questionnaire in 2005 (response rate 44%). The cohort participants recapitulated well the distance learning student body at STOU and share certain geo-demographic attributes with the general Thai population (mean age was 29 years in 2005, slightly more than half were females, half resided in urban areas).[21, 22] The baseline questionnaire gathered data on a wide range of topics including age, sex, income, marital status, health status, doctor-diagnosed diseases, health-risk behaviors including smoking and drinking, social capital, and history of injury.

Mortality data

The completeness of death registration in Thailand was 86% from 1950-2000[10], but over the last decade coverage improved to 95%.[23] A powerful feature of our study is that all cohort members have provided their Thai Citizen ID number enabling detection and analysis of deaths into the future. These confidential ID numbers were safeguarded and stored at STOU in a secure office on the main campus with 24-hour guards on patrol. The working files of these data were de-identified and no individual information will be released or displayed in any format. To detect

For peer review only

deaths, the Bangkok TCS team periodically matched the cohort against national death records from the Ministry of Interior using the Citizen ID number. At a later stage the Thai Ministry of Public Health expanded these death records by adding the standard International Classification of Diseases (ICD-10)[24] to identify causes of death.

Up until March 2010, there were a total of 580 deaths among the Thai Cohort Study participants. According to the ICD-10 codes, there were 376 deaths from non-injury causes including ill-defined causes of death. For the purpose of this study, these will not be broken down and will be designated as 'other deaths'. For our injury-focused death analysis, there were 204 deaths from external causes, including 84 deaths from transport accidents. Among the 120 non-transport injury deaths, there were 35 deaths from miscellaneous external causes, 10 deaths from intentional self-harm, 30 deaths from assault, and 45 deaths from 'unspecified events of undetermined intent'.

Exposures and confounders

In our analysis, exposures of interest and potential confounders from the 2005 baseline questionnaire included the following geo-demographic variables: age (4 categories), sex, marital status (married, not married, divorced/ widowed), personal monthly income (≤3000 Baht, 3001-7000, 70001-10000, 10001-20000, >20000), regions (Central/East, Bangkok, North, Northeast, South), and lifecourse urbanisation (residence at age 12 years old and at baseline: rural-rural, rural-urban, urban-rural, urban-urban). As well, a history of injury in the past year was reported at 2005 baseline, including the frequency and location of injuries reported.

Also analysed as exposures of interest were certain social covariates, several health states, and important health-risk behaviours. These behaviours included smoking and alcohol drinking which have been shown to be independently associated with injury.[25, 26] Smoking status includes never, current, and former and alcohol status includes never, occasional, regular, and former. In addition, at baseline cohort members were asked "in the last year have you driven a motor vehicle after consuming 3 or more glasses of alcohol" (ie drink driving). Other health-related attributes included self-assessed health and chronic metabolic or cardiovascular disorders (e.g. diabetes, hypertension). A history of doctor-diagnosed depression has previously been shown to be an injury risk[18, 27] and is also included in the model. Social capital was dichotomoised for analyses (low or not low) in three domains: trust (whether people can be trusted), support (from family, friends, colleagues), and interaction (with family, friends, neighbours).

For peer review only

Data processing and statistical analysis

Questionnaire responses were digitised by optical scanning and subsequently edited using Thai Scandevet, SQL and SPSS software. For analysis we used Stata version 12. Individuals with missing data for given analyses were excluded (<5%), so totals vary slightly according to available information. We described the distribution of deaths by demographic, social, and health covariates, by cause of death, and by types of injury (transport and non-transport injuries). In addition, we presented death rates per 10,000 person-years of exposure. We then used age-sex adjusted multinomial logistic regression linking the above covariates to three possible outcomes: alive (reference), deaths from other causes, and deaths from injury (study outcome). The final analytical model mutually adjusted for all covariates and reported Adjusted Odds Ratios (AOR) and 95% Confidence Intervals.

Results

Among Thai cohort members at baseline in 2005 (Table 1), about one third of the cohort were less than 30 years old, slightly more than half were female, about 40% reported monthly income of less than 7000 Baht per month (175 US$ in 2005), 18% lived in Bangkok, and about half were urban residents. Injury deaths were more likely to affect males (73.7%, 15 vs 23 per 10000 person-years for transport vs non-transport injuries). Also notable, injury deaths were disproportionately frequent in the Southern region (20.6%, 12 vs 25 per 10000 person-years for transport vs non-transport injuries).

For social and health attributes (Table 2), a history of ever drink driving in the past year was more common among injury deaths (42.4% compared to 26.5% for other deaths or 25.4% for alive) and notably higher for transport than non-transport injuries (52.9% vs 35.0%). Cohort members who died from non-injury related causes were twice as likely to have reported poor self-assessed health at baseline and three times as likely to have reported metabolic and cardiovascular chronic conditions. Cohort members who died from injury reported higher rates of ever having been doctor-diagnosed depression (6.9% compared to 3.4% among non-deaths). As well, a history of depression was much more frequent for those who died from non-transport injuries than for transport injuries (34 vs 13 per 10000 person-years). At baseline in 2005, about 20% of cohort members overall reported injury at least once in the past year compared to 33.3% of cohort participants who died from transport injury.

For peer review only

In addition to analysing by injury types, we also tabulated the death rates according to the International Classification of Diseases (Table 3). Within transport injury mortality, rates per 10000 person-years for motorcycle riders and car occupants were 1.6 and 1.7, respectively. There were also 4.5 per 10000 person-years who died in unspecified motor vehicles. Among non-transport injury deaths, the rate per 10000 person-years of assault by firearm discharge was 2.5 with an additional 1.5 deaths from firearm discharge with undetermined intent. Deaths from drowning and submersion were 1.3 per 10000 person-years. Intentional self-harm deaths included self-poisoning and hanging-strangulation-suffocation with death rates of 0.3 and 0.8 per 10000 person-years, respectively.

To examine predictors of injury deaths (Table 4), we used multinomial logistic regression with three outcome categories: alive (reference), non-injury deaths, and injury deaths (study outcome). Highlighted in bold were results that were statistically significant at p<0.05. In the first column of odds ratios, the results are adjusted for age and sex; in the second column the odds ratios are adjusted for all covariates. All odds ratios compare the odds of injury death to the odds of staying alive.

We first calculate age-sex adjusted odds ratios for each potential exposure and for covariates. In the age-sex adjusted mode, being younger than 30 years, being male, residing in the South, currently smoking, drink driving in the past year, ever having been diagnosed for depression, having low social support, injury incidence in the year preceding baseline, and reported road injury in 2005, all were associated with injury mortality.

We then proceed to multivariate analysis (Table 4). After mutually adjusting for all tabulated covariates, statistically significant predictors of injury mortality were being male (Adjusted Odds Ratio, AOR 3.87), residing in the Southern areas (AOR 1.71), being a current smoker (AOR 1.56), history of drink driving (AOR 1.49) and ever having been diagnosed for depression (AOR 1.91). Other covariates such as being young, having low social support, and reporting road injury in the past year at baseline had predictive AORs ranging from 1.4 to 1.6, but these substantive estimates were not statistically significant for overall injury. Further investigation into types of injury (data not shown) revealed that, younger age was a strong predictor for transport injury deaths (AOR 4.12, 95% 1.03-16.5) and low social support for non-transport injury deaths (AOR 1.64, 95% 1.04-2.59).

For peer review only

In marked contrast to the injury deaths, cohort members who died from other causes had different sets of statistically significant risks at 2005 baseline which included older age, residing in Bangkok, reporting poor self-assessed health, and having metabolic or cardiovascular chronic conditions (data not shown). The only risk factor that non-injury deaths have in common with injury deaths was being a current smoker.

Discussion

This study is embedded in an overarching investigation of health-risk transition in Thailand, where similar to middle-income Southeast Asian countries; injury has been and still is a major population health burden. We make use of our large cohort of adults to investigate risk factors associated with injury mortality, linking deaths over five years (2005-2010) to individual characteristics and social and health attributes recorded at baseline (2005). The results revealed that injuries constituted almost one third of all deaths in the cohort, and some 40% of those were from transport and nearly 60% were non-transport injuries. Connecting to baseline information provided five years earlier, we identified epidemiologically and statistically significant predictors of injury mortality. These predictors included certain geo-demographic characteristics (male sex, Southern residents), health risk behaviours (smoking and history of drink driving), and adverse health diagnoses (depression).

Our injury mortality findings provide further information adding to our previous knowledge on risk factors associated with injury morbidity in the cohort, especially being male, having a history of depression, and drink driving.[18-20] The effect of smoking on injury was also shown in other studies after controlling for covariates including alcohol drinking. Plausible explanations include accidental fire hazard and distraction or inattention for road traffic hazards.[25, 26] Non-transport injury deaths presented here reflected the current political situation with violence in the Southern part of Thailand since 2004.[28, 29] For covariates with substantial (but not statistically significant) point estimates of overall injury death (young age, low social support, history of road injury), AORs ranged from 1.4-1.6.

Our results support other published international information on risk factors related to injury mortality, showing young males and drunk drivers at high risk of road crash death.[30, 31] A systematic review on alcohol consumption and collision risk concluded that there is no safe

For peer review only

level of drinking and even less than two drinks per occasion can almost double the odds of most types of injury.[30]

Our study also found social capital to be protective against injury mortality, supporting previous research.[32-34] Indeed, we found low social support to be a predictor of non-transport injury mortality. But social support did not relate to transport injury mortality. Despite the relatively small number of deaths in our study, we found that low social support was particularly associated with intentional self-harm and assault but these associations were not statistically significant.

The advantage of our study is its large participation by Thai adults who completed a comprehensive 20-page baseline questionnaire in 2005, reporting on a wide array of social and health characteristics. This provided a platform for investigation of causal pathways and elimination of many confounders. While our cohort members share similar distribution of sex, modest income, and geographical residence with the general Thai population. However, all cohort members completed high school education which facilitated their ability to respond to our detailed questionnaire. We also noted that a few (<5%) cohort members were 'missing' data for variables used in various models but this problem was not numerically significant. We acknowledge that our study captured the 5-year mortality rate after the baseline questionnaire in 2005 and the number of deaths was relatively small. However, the citizen IDs provided by cohort members at the baseline can be used to monitor patterns of mortality into the future and eventually a full account of cohort mortality will be possible.

Our research contributes to limited longitudinal evidence linking risk factors to injury death and is one of the first studies in middle-income Southeast Asia. We have achieved our aim of identifying vulnerable population subgroups at risk of injury deaths. As well, we complemented routinely collected administration death registration with a longitudinal health assessment providing information on individual, social, and health history factors. This information is invaluable in yielding insight into certain risk traits such as being a young male, reporting and having a past medical diagnosis of depression, which could inform injury prevention policies and strategies suitable for transitional countries with limited resources and changing patterns of mortality.

For peer review only

Contributors

VY conceptualized, analysed and drafted the manuscript. JB-G, RM, CB provided inputs to improve the draft. SS and AS designed and executed the Thai Cohort Study. AS provided comments and guidance on various revisions. All authors read and approved the final manuscript.

Author footnote

The Thai Cohort Study Team

Thailand: Jaruwan Chokhanapitak, Suttanit Hounthasarn, Suwanee Khamman, Daoruang Pandee, Suttinan Pangsap, Tippawan Prapamontol, Janya Puengson, Sam-ang Seubsman, Boonchai Somboonsook, Nintita Sripaiboonkij, Pathumvadee Somsamai, Duangkae Vilainerun, Wanee Wimonwattanaphan, Tewarit Somkotra, Benjawan Tawatsupa, Wimalin Rimpeekool.

Australia: Chris Bain, Emily Banks, Cathy Banwell, Bruce Caldwell, Gordon Carmichael, Tarie Dellora, Jane Dixon, Sharon Friel, David Harley, Matthew Kelly, Tord Kjellstrom, Lynette Lim, Anthony McMichael, Tanya Mark, Penny Haora, Adrian Sleigh, Lyndall Strazdins, Susan Jordan, Roderick McClure, Janneke Berecki-Gisolf, Vasoontara Yiengprugsawan.

Acknowledgments

We thank the staff at Sukhothai Thammathirat Open University (STOU) who assisted with student contact and the STOU students who are participating in the cohort study. We also thank Dr Bandit Thinkamrop and his team from Khon Kaen University for support on initial data processing. As well we would like thank the Thai Ministry of Interior and Ministry of Public Health for mortality investigation and cause of death data linkage. Matthew Kelly and Peter Sbirakos provided language editorial support.

Funding

This study was supported by the International Collaborative Research Grants Scheme with joint grants from the Wellcome Trust UK (GR071587MA) and the Australian National Health and Medical Research Council (268055), and as a global health grant from the NHMRC (585426).

Ethical approval

Informed written consent was obtained from all participants. All students were advised that they could withdraw, or not participate, without any effect on their academic progress. The questionnaires never sought sensitive personal information and no biological samples were taken. Ethics approval was obtained from Sukhothai Thammathirat Open University Research and Development Institute (protocol 0522/10) and the Australian National University Human Research Ethics Committee (protocols 2004/344 and 2009/570).

Competing interests

None declared.

Data sharing statement

Data are available through a data access agreement which includes guarantees regarding ethical conduct and scientific quality of any proposed analyses and publications. Anyone wanting access should contact Professor Adrian Sleigh or Associate Professor Sam-ang Seubsman (Thai Cohort Study Principal Investigators).

For peer review only

Table 1 Distribution of mortality by baseline social and geo-demographic attributes, Thai Cohort Study

Cohort attributes	Vital status by attributes %					Incidence per 10000 person-years	
	Alive (86457)	Other deaths (376)	Injury deaths (204)	Injury deaths (204)		Transport (84)	Non-transport (120)
				Transport (84)	Non-transport (120)		
**Age groups in years:**							
18-29	31.4	27.7	27.9	20.2	25.7	6	15
30-39	53.6	29.5	59.3	69.1	51.4	12	14
40-49	12.5	25.8	9.3	6.0	17.1	5	13
≥50	2.4	17.0	3.4	4.8	5.7	19	14
**Sex:**							
Males	45.2	65.6	73.7	69.4	76.7	15	23
**Marital status:**							
Married	38.8	50.1	35.0	30.9	37.7	8	14
Not married	56.8	41.9	60.1	64.3	57.1	11	14
Divorced/widowed	4.4	8.0	4.9	4.8	5.0	11	16
**Personal monthly income:**							
≤3000 Baht	11.0	14.9	14.6	11.9	16.5	10	20
3001-7000	30.9	22.4	33.2	39.3	28.7	13	13
7001-10000	23.3	16.6	23.6	26.2	21.7	11	13
10001-20000	24.2	28.7	20.6	16.7	23.5	7	13
>20000	10.5	17.4	8.0	6.0	9.6	6	12
**Regions:**							
Central/East	30.7	30.7	24.9	23.8	24.2	8	11
Bangkok	17.2	18.7	10.7	9.4	11.7	5	9
North	18.2	22.7	21.0	23.5	19.2	13	15
Northeast	20.9	21.6	23.4	25.9	21.7	12	14
South	13.0	12.3	20.6	16.7	23.3	12	25
**Lifecourse residence:**							
Rural-rural	43.3	42.3	46.6	38.1	52.5	8	17
Rural-urban	31.5	25.0	16.7	28.6	30.8	9	13
Urban-rural	4.2	6.9	4.4	7.1	2.5	16	8
Urban-urban	19.7	22.3	16.7	22.6	12.5	11	9

For peer review only

13

Table 2 Mortality by baseline health-risk behaviours and states, social attributes, and history of injury, Thai Cohort Study

Social and health attributes	Vital status by attributes %					Incidence per 10000 person-years	
	Alive (86457)	Other deaths (376)	Injury deaths (204)	Injury deaths (204) Transport (84)	Non-transport (120)	Transport (84)	Non-transport (120)
**Health-risk attributes**							
**Smoking:**							
Never	72.3	51.1	57.5	66.3	51.3	9	10
Current	10.0	19.8	24.0	21.7	25.6	21	35
Former	15.8	26.1	15.5	9.6	19.7	6	17
**Alcohol drinking:**							
Never	26.5	22.9	19.8	21.4	18.6	8	10
Occasional	59.8	49.5	60.9	61.9	60.2	10	14
Regular	4.8	9.7	7.9	9.5	6.8	19	19
Stop	8.9	18.0	11.4	7.1	14.4	8	22
**Ever drink driving in past year:**							
Yes	25.4	26.5	42.4	52.9	35.0	20	19
Do not usually drive	8.8	8.2	8.8	9.4	8.3	10	13
**Health and social attributes**							
**Self-assessed health:**							
Poor or very poor	4.6	8.8	3.9	2.4	5.0	7	16
**Chronic conditions:**							
Yes	12.5	29.3	10.8	13.1	9.2	10	13
**Doctor diagnosed depression:**							
Yes	3.4	5.9	6.9	4.8	8.3	13	34
**Social capital:**							
Low trust	38.2	36.9	34.5	35.4	33.9	9	12
Low support	25.5	33.2	20.1	22.6	18.3	9	20
Low interaction	23.3	25.5	28.9	22.6	33.3	9	10
**Injury reported in 2005**							
**Number of injuries:**							
At least once	20.2	29.5	27.9	33.3	24.2	16	16
**Location of injury:**							
Home	5.3	7.1	5.4	5.9	5.0	10	14
Road	5.9	4.8	11.7	16.5	8.3	25	20
Work	3.9	6.4	5.4	9.5	2.5	11	13

For peer review only

Table 3 Injury mortality by International Classification of Diseases (ICD-10), Thai Cohort Study

Types of injury deaths	Number of deaths	Rate per 10000 person-years
**Transport injuries**		
V01-V09 Pedestrian	2	0.2
V20-V29 Motorcycle rider	14	1.6
V40-V49 Car occupant	15	1.7
V50-59 Occupant of pick-up truck or van	3	1.0
V80-V89 Other land transport accident	9	0.1
V89.2 Person injured in unspecified motor vehicle	39	4.5
V90-V94 Water transport	1	0.1
V95-V97 Air and space transport	1	0.1
**Non-transport injuries**		
W00-W19 Falls	2	0.3
W65-W74 Drowning and submersion	10	1.3
W75-W84 Other threats to breathing	1	0.1
W87 Exposure to electric current	2	0.3
X00-X09 Exposure to smoke, fire and flames	2	0.3
X33 Victim of lighting	1	0.1
X38 Victim of flood	1	0.1
X58-X59 Exposure to other unspecified factors	16	2.1
X60-X84 Intentional self-harm		
X65 Intentional self-poisoning	3	0.3
X70 Intentional self-harm by hanging, strangulation and suffocation	7	0.8
X85-Y09 Assault		
X95 Assault by unspecified firearm discharge	22	2.5
X99 Assault by sharp object	3	0.3
Y99 Assault by other unspecified means	5	0.6
Y10-Y34 Event of undetermined intent		
Y18 Poisoning by and exposure to pesticides	1	0.1
Y20 Hanging, strangulation and suffocation	3	0.3
Y22-Y24 Firearm discharge, undetermined intent	13	1.5
Y25 Contact with explosive material	1	0.1
Y28-Y29 Contact with sharp of blunt object	4	0.5
Y34 Unspecified event, undetermined intent	23	2.6

For peer review only

Table 4 Age-sex adjusted and multivariate predictors of injury mortality, Thai Cohort Study

2005 baseline covariates	Multinomial* Adjusted Odds Ratios [95% CI]			
	Age-sex adjusted		Multivariate**	
	Alive	Injury death	Alive	Injury death
**Geo-demographic covariates**				
**Age groups in years:** 20-29		**1.45 [1.06-1.99]**		1.51 [0.99-2.32]
30-39	*Ref*		*Ref*	
40-49		0.76 [0.45-1.28]		0.99 [0.54-1.83]
≥50		1.29 [0.59-2.84]		1.65 [0.61-4.43]
**Sex** *(female)*:	*Ref*		*Ref*	
Male		**3.69 [2.69-5.07]**		**3.87[2.39-6.26]**
**Marital status** *(married)*:	*Ref*		*Ref*	
Not married		1.09 [0.77-1.56]		1.11 [0.73-1.69]
Divorced/widowed		1.55 [0.80-3.02]		1.47 [0.69-3.14]
**Personal monthly income:**				
≤3000Baht		1.24 [0.80-1.93]		1.10 [0.48-1.53]
3001-7000		1.25 [0.89-1.75]		1.15 [0.78-1.69]
7001-20000		0.81 [0.46-1.43]		0.83 [0.43-1.61]
>20000	*Ref*		*Ref*	
**Regions** (*Central/East*):	*Ref*		*Ref*	
Bangkok		0.85 [0.51-1.41]		0.86 [0.48-1.53]
North		1.38 [0.92-2.08]		1.27 [0.80-2.01]
Northeast		1.29 [0.87-1.93]		0.98 [0.61-1.56]
South		**1.96 [1.30-2.97]**		**1.71 [1.05-2.79]**
**Lifecourse residence** *(rural-rural)*:	*Ref*		*Ref*	
Urban-rural		0.91 [0.66-1.27]		0.97 [0.65-1.44]
Rural-urban		1.03 [0.52-2.04]		1.32 [0.73-1.44]
Urban-urban		0.87 [0.59-1.29]		1.12 [0.71-1.77]
**Health and social covariates**				
**Smoking** *(never)*:	*Ref*		*Ref*	
Current		**1.70 [1.07-2.05]**		**1.56 [1.03-2.37]**
Former		0.77 [0.45-1.32]		0.74 [0.47-1.18]
**Alcohol drinking** *(never)*:	*Ref*		*Ref*	
Occasional		0.87 [0.60-1.27]		0.40 [0.12-1.30]
Regular		1.06 [0.58-1.95]		0.38 [0.10-1.40]
Stop		1.08 [0.64-1.84]		0.63 [0.18-2.15]
**Drink driving past year** *(never)*:	*Ref*		*Ref*	
Yes		**1.50 [1.07-2.12]**		**1.49 [1.01-2.20]**
**Self-assessed health** *(good)*:	*Ref*		*Ref*	
Poor or very poor		1.09 [0.58-2.07]		0.75 [0.32-1.71]
**Depression** *(no)*:	*Ref*		*Ref*	
Yes		**2.15 [1.25-3.71]**		**1.91 [1.00-3.69]**
**Chronic illness** *(no)*:	*Ref*		*Ref*	
Yes		0.80 [0.50-1.2]		0.84 [0.50-1.44]
**Social capital:**				
Low social trust (*ref: not low*)		084 [0.60-1.19]		0.90 [0.76-1.07]
Low social support (*ref*: not low)		1.29 [0.58-2.84]		1.37 [0.96-1.96]
Low social interaction (*ref*: not low)		0.86 [0.64-1.15]		0.77 [0.50-1.20]
**Injury reported in 2005**				
**Injuries in the past year** *(no)*:	*Ref*		*Ref*	
At least once		**1.41 [1.04-1.92]**		1.12 [0.70-1.82]
**Location of injury:**				
Home (*ref: no*)		1.13 [0.62-2.08]		1.19 [0.56-2.55]
Road (*ref: no*)		**1.81 [1.17-2.79]**		1.58 [0.87-2.84]
Work (*ref: no*)		1.22 [0.66-2.25]		1.15 [0.54-2.44]

*multinomial logistic regression compares the odds of injury deaths to the odds of remaining alive by predictor covariate category values, after adjusting for age-sex or other covariates.
**mutually adjusted for all predictor covariates presented in this table

For peer review only

References

1. Stevenson M. Measuring the global burden of road traffic injury: implications for low-income and middle-income countries. Inj Prev. 2009; 15:1-2.
2. Lim SS, Vos T, Flaxman AD, et al. A comparative risk assessment of burden of disease and injury attributable to 67 risk factors and risk factor clusters in 21 regions, 1990-2010: a systematic analysis for the Global Burden of Disease Study 2010. Lancet. 2012; 380:2224-60.
3. WHO. Global status report on road safety 2013: supporting a decade of action. Geneva: World Health Organization; 2013.
4. WHO. World report on road traffic injury prevention. Geneva: World Health Organization; 2004.
5. Chandran A, Hyder AA, Peek-Asa C. The global burden of unintentional injuries and an agenda for progress. Epidemiol Rev. 2010; 32:110-20.
6. WHO. Global status report on violence prevention. Geneva: World Health Organization; 2014.
7. Bhalla K, Harrison J, Abraham J, Borse NN, Lyons R, Boufous S, et al. Data sources for improving estimates of the global burden of injuries: call for contributors. PLoS Med. 2009; 6 e1 doi: 10.1371/journal.pmed.1000001.
8. Bhalla K, Harrison JE, Fingerhut LA, et al. The global injury mortality data collection of the Global Burden of Disease Injury Expert Group: a publicly accessible research tool. Int J Inj Contr Saf Promot. 2011; 18:249-53.
9. Baker SP, Li G. Epidemiologic approaches to injury and violence. Epidemiol Rev. 2011; 34:1-3.
10. Mathers CD, Fat DM, Inoue M, et al. Counting the dead and what they died from: an assessment of the global status of cause of death data. Bull World Health Organ. 2005; 83:171.
11. Ditsuwan V, Veerman LJ, Barendregt JJ, et al. The national burden of road traffic injuries in Thailand. Popul Health Metr. 2011; 9:2; doi: 10.1186/478-7954-9-2.
12. Chadbunchachai W, Suphanchaimaj W, Settasatien A, et al. Road traffic injuries in Thailand: current situation. J Med Assoc Thai. 2012; 95 Suppl 7:S274-81.
13. Choprapawon C, Porapakkham Y, Sablon O, et al. Thailand's national death registration reform: verifying the causes of death between July 1997 and December 1999. Asia Pac J Public Health. 2005; 17:110-6.
14. Tangcharoensathien V, Faramnuayphol P, Teokul W, et al. A critical assessment of mortality statistics in Thailand: potential for improvements. Bull World Health Organ. 2006; 84:233-8.
15. Rao C, Porapakkham Y, Pattaraarchachai J, et al. Verifying causes of death in Thailand: rationale and methods for empirical investigation. Popul Health Metr. 2010; 8:11.
16. Porapakkham Y, Rao C, Pattaraarchachai J, P et al. Estimated causes of death in Thailand, 2005: implications for health policy. Popul Health Metr. 2010; 8:14.
17. Pattaraarchachai J, Rao C, Polprasert W, et al. Cause-specific mortality patterns among hospital deaths in Thailand: validating routine death certification. Popul Health Metr. 2010; 8:12.
18. Yiengprugsawan V, Stephan K, McClure R, et. al. Risk factors for injury in a national cohort of 87,134 Thai adults. Public Health. 2012; 126:33-9.
19. Stephan K, Kelly M, McClure R, et. al. Distribution of transport injury and related risk behaviours in a large national cohort of Thai adults. Accid Anal Prev. 2011; 43:1062-7.
20. Tawatsupa B, Yiengprugsawan V, Kjellstrom T, Berecki-Gisolf J, Seubsman SA, Sleigh A. Association between heat stress and occupational injury among Thai workers: findings of the Thai Cohort Study. Ind Health. 2013; 51:34-46.
21. Sleigh AC, Seubsman SA, Bain C. Cohort profile: The Thai Cohort of 87,134 Open University students. Int J Epidemiol. 2008; 37:266-72.
22. Seubsman SA, Yiengprugsawan V, Sleigh AC, the Thai Cohort Study T. A large national Thai Cohort Study of the Health-Risk Transition based on Sukhothai Thammathirat Open University students. ASEAN J Open Distance Learn. 2012; 4:58-69.
23. Prasartkul P, Vapattanawong P. The completeness of death registration in Thailand: evidence from demographic surveillance system of the kanchanaburi project. World Health Popul. 2006; 8:43-51.
24. WHO. International Statistical Classification of Diseases and Related Health Problems, 10th Revision. http://apps.who.int/classifications/icd10/browse/2010/en: World Health Organization.; 2011.
25. Leistikow BN, Martin DC, Jacobs J, et. al. Smoking as a risk factor for accident death: a meta-analysis of cohort studies. Accid Anal Prev. 2000; 32:397-405.
26. Wen CP, Tsai SP, Cheng TY, et. al. Excess injury mortality among smokers: a neglected tobacco hazard. Tobacco control. 2005; 14 Suppl 1:i28-32.

For peer review only

27. Patten SB, Williams JV, Lavorato DH, et. al. Major depression and injury risk. Can J Psychiatry. 2010; 55:313-8.
28. Bonura C. Indeterminate geographies of political violence in Southern Thailand. Alternatives. 2008; 33:383-412.
29. McCargo D. Rethinking Thailand's southern violence - Introduction. Crit Asian Stud. 2006; 38:3-10.
30. Taylor B, Irving HM, Kanteres F, Room R, Borges G, Cherpitel C, et al. The more you drink, the harder you fall: a systematic review and meta-analysis of how acute alcohol consumption and injury or collision risk increase together. Drug Alcohol Depend. 2010; 110:108-16.
31. Connor J, Norton R, Ameratunga S, et. al. The contribution of alcohol to serious car crash injuries. Epidemiology. 2004; 15:337-44.
32. Aida J, Kondo K, Hirai H, et. al. Assessing the association between all-cause mortality and multiple aspects of individual social capital among the older Japanese. BMC Public Health. 2011; 11:499.
33. Hyyppa MT, Maki J, Impivaara O, et. al. Individual-level measures of social capital as predictors of all-cause and cardiovascular mortality: a population-based prospective study of men and women in Finland. Eur J Epidemiol. 2007; 22:589-97.
34. Muennig P, Cohen AK, Palmer A, et. al. The relationship between five different measures of structural social capital, medical examination outcomes, and mortality. Soc Sci Med. 2013; 85:18-26.

For peer review only

Predictors of injury mortality: findings from a large national cohort in Thailand

Vasoontara Yiengprugsawan[1§], Janneke Berecki-Gisolf[2], Roderick McClure[2], Christopher Bain[3], Sam-ang Seubsman[1,4], Adrian C Sleigh[1]

[1] National Centre for Epidemiology and Population Health, The Australian National University, Canberra, Australia
[2] Monash Injury Research Institute, Monash University, Melbourne, Australia
[3] Genetics and Population Health Division, Queensland Institute of Medical Research, Brisbane, Queensland, Australia
[4] School of Human Ecology, Sukhothai Thammathirat Open University, Nonthaburi, Thailand

[§] **Correspondence to:** Dr. Vasoontara Yiengprugsawan, The Australian National University, College of Medicine, Biology and Environment, Research School of Population Health, National Centre for Epidemiology and Population Health, Building 62, Mills Rd, Acton 2601, Canberra, Australia Tel: +61 2 6125 2378; Fax: +61 2 6125 0740
Email vasoontara.yieng@anu.edu.au; vasoontara.yieng@gmail.com

For peer review only

Predictors of injury mortality: findings from a large national cohort in Thailand

Abstract

Objective: to present predictors of injury mortality by types of injury and by pre-existing attributes or other individual exposures identified at baseline.

Design: 5-year prospective longitudinal study

Setting: Contemporary Thailand (2005-2010), a country undergoing epidemiological transition

Participants: Data derived from a research cohort of 87,037 distance-learning students enrolled at Sukhothai Thammathirat Open University (STOU) residing nationwide.

Measures: Cohort members completed a comprehensive baseline mail-out questionnaire in 2005 reporting geo-demographic, behavioural, health, and injury data. These responses were matched with national death records using the Thai Citizen ID number. Age-sex adjusted multinomial logistic regression was used to calculate odds ratios linking exposure variables collected at baseline to injury deaths over the next five years.

Results: Statistically significant predictors of injury mortality were being male (Adjusted Odds Ratio 3.87, 95% Confidence Interval 2.39-6.26), residing in the Southern areas (AOR 1.71, 1.05-2.79), being a current smoker (1.56, 1.03-2.37), history of drink driving (AOR 1.49,1.01-2.20) and ever having been diagnosed for depression (AOR 1.91, 1.00-3.69). Other covariates such as being young, having low social support, and reporting road injury in the past year at baseline had moderately predictive AORs ranging from 1.4 to 1.6 but were not statistically significant.

Conclusions: We complemented national death registration with longitudinal data on individual, social, and health attributes. This information is invaluable in yielding insight into certain risk traits such as being a young male, history of drink driving, and history of depression. Such information could be used to inform injury prevention policies and strategies.

For peer review only

Article summary:

Article focus

- Injury is a population health burden in transitional low and middle-income Southeast Asia.
- We investigated a large national cohort of Thai adults for predictors of injury mortality including geo-demographic, social, and health attributes recorded at baseline.

Key messages:

- Injuries constituted almost one third of all deaths in the cohort, and some 40% of those were from transport and nearly 60% were non-transport injuries.
- These injury mortality observations add to our previous Thai work on injury morbidity, highlighting the overall risks, especially depression, male sex, and drink driving.
- The link of the Southern Thai region to non-transport injury deaths most likely reflects the political violence which covers the period since 2004.

Strengths and limitations of this study:

- The advantage of our study is its large size, longitudinal design, and comprehensive baseline information. This provides a platform for identification of risks, elimination of confounders, and exploration of causal pathways.
- This study captured the 5-year mortality rate in a generally young adult cohort. Thus there were relatively few deaths.
- Citizen IDs provided at baseline will enable us to monitor patterns of cohort mortality into the future.

For peer review only

Introduction

Injury remains a major public health challenge worldwide; causing one tenth of global mortality with a heavy burden in developing countries.[1, 2] According to the World Health Organization, at least 1.2 million people are killed from road crashes and an estimated 50 million are injured on roads worldwide each year.[3, 4] Violence and non-transport injuries also accounted for more than 1.3 million deaths and many suffered from serious physical and mental consequences.[5, 6]

Most national injury prevention policies have been introduced in high-income countries. Unfortunately, very few low and middle-income countries have been able to develop such policies due to lack of resources and limited availability of quality injury mortality data.[7, 8] In particular, many developing countries still face the challenges of accurately identifying causes of death from routinely collected national civil registration and vital statistics systems while other sources of data such as police reports and hospital records are never comprehensive and lead to underreporting bias if relied upon as a main source of injury mortality data. Reliable cause-of-death data are important because they enable monitoring of the epidemiological occurrence and public health effects at the population level.[9, 10]

Throughout middle-income Southeast Asia, including Thailand, injury continues to be one of the top ten causes of death.[11, 12] In past decades, Thailand has reformed administrative records to improve the coverage and quality of cause-specific mortality data.[13, 14] Eight years ago, Thailand began to study the ill-defined causes of death by using verbal autopsies and these revealed that besides a high proportion of transport-related deaths, a number of other deaths which were initially recorded as non-specific causes turned out to be suicides, assaults, and drowning.[15-17] These findings shed light on the importance of non-transport injuries in addition to the burden of transport injuries.

This study is based on a large national cohort in Thailand which has been followed to investigate health-risk transitions of Thai adults since 2005. The cohort database includes comprehensive information on individual characteristics, social demography, health behaviours and specific diseases, as well as history of injuries. Our previous research based on this cohort has examined risk factors associated with injury morbidity.[18-20] Now successful mortality data linkage

through the Thai Ministry of Interior and Ministry of Public Health allows us to analyse injury-related deaths among the cohort over the first five years (2005-10).

Informed by our earlier research on injury morbidity, and by related published information, this study has investigated injury in more depth using mortality as the outcome. Our study linked cohort outcomes (survival, non-injury death, injury death) to an array of relevant exposures recorded at baseline including geo-demographic attributes, social covariates, health and psychological states, and health-risk behaviours. This analysis is both prospective and cohort-based and fills an important gap regarding our knowledge of injury risks as an emerging public health problem in a middle-income Asian country going through the health-risk transition.

Methods

Study population and data collection

This analysis is part of the overarching Thai Cohort Study (TCS), an ongoing epidemiological investigation of changing patterns of health risks and outcomes. Data derive from a research cohort of 87,037 distance-learning adult students enrolled at Sukhothai Thammathirat Open University (STOU), who resided all over Thailand and completed the baseline comprehensive mail-out health questionnaire in 2005 (response rate 44%). The cohort participants recapitulated well the distance learning student body at STOU and share certain geo-demographic attributes with the general Thai population (mean age was 29 years in 2005, slightly more than half were females, half resided in urban areas).[21, 22] The baseline questionnaire gathered data on a wide range of topics including age, sex, income, marital status, health status, doctor-diagnosed diseases, health-risk behaviors including smoking and drinking, social capital, and history of injury.

Mortality data

The completeness of death registration in Thailand was 86% from 1950-2000[10], but over the last decade coverage improved to 95%.[23] A powerful feature of our study is that all cohort members have provided their Thai Citizen ID number enabling detection and analysis of deaths into the future. These confidential ID numbers were safeguarded and stored at STOU in a secure office on the main campus with 24-hour guards on patrol. The working files of these data were de-identified and no individual information will be released or displayed in any format. To detect

For peer review only

deaths, the Bangkok TCS team periodically matched the cohort against national death records from the Ministry of Interior using the Citizen ID number. At a later stage the Thai Ministry of Public Health expanded these death records by adding the standard International Classification of Diseases (ICD-10)[24] to identify causes of death.

Up until March 2010, there were a total of 580 deaths among the Thai Cohort Study participants. According to the ICD-10 codes, there were 376 deaths from non-injury causes including ill-defined causes of death. For the purpose of this study, these will not be broken down and will be designated as 'other deaths'. For our injury-focused death analysis, there were 204 deaths from external causes, including 84 deaths from transport accidents. Among the 120 non-transport injury deaths, there were 35 deaths from miscellaneous external causes, 10 deaths from intentional self-harm, 30 deaths from assault, and 45 deaths from 'unspecified events of undetermined intent'.

Exposures and confounders

In our analysis, exposures of interest and potential confounders from the 2005 baseline questionnaire included the following geo-demographic variables: age (4 categories), sex, marital status (married, not married, divorced/ widowed), personal monthly income (≤3000 Baht, 3001-7000, 70001-10000, 10001-20000, >20000), regions (Central/East, Bangkok, North, Northeast, South), and lifecourse urbanisation (residence at age 12 years old and at baseline: rural-rural, rural-urban, urban-rural, urban-urban). As well, a history of injury in the past year was reported at 2005 baseline, including the frequency and location of injuries reported.

Also analysed as exposures of interest were certain social covariates, several health states, and important health-risk behaviours. These behaviours included smoking and alcohol drinking which have been shown to be independently associated with injury.[25, 26] Smoking status includes never, current, and former and alcohol status includes never, occasional, regular, and former. In addition, at baseline cohort members were asked "in the last year have you driven a motor vehicle after consuming 3 or more glasses of alcohol" (ie drink driving). Other health-related attributes included self-assessed health and chronic metabolic or cardiovascular disorders (e.g. diabetes, hypertension). A history of doctor-diagnosed depression has previously been shown to be an injury risk[18, 27] and is also included in the model. Social capital was dichotomoised for analyses (low or not low) in three domains: trust (whether people can be trusted), support (from family, friends, colleagues), and interaction (with family, friends, neighbours).

For peer review only - http://bmjopen.bmj.com/site/about/guidelines.xhtml

Data processing and statistical analysis

Questionnaire responses were digitised by optical scanning and subsequently edited using Thai Scandevet, SQL and SPSS software. For analysis we used Stata version 12. Individuals with missing data for given analyses were excluded (<5%), so totals vary slightly according to available information. We described the distribution of deaths by demographic, social, and health covariates, by cause of death, and by types of injury (transport and non-transport injuries). In addition, we presented death rates per 10,000 person-years of exposure. We then used age-sex adjusted multinomial logistic regression linking the above covariates to three possible outcomes: alive (reference), deaths from other causes, and deaths from injury (study outcome). The final analytical model mutually adjusted for all covariates and reported Adjusted Odds Ratios (AOR) and 95% Confidence Intervals.

Results

Among Thai cohort members at baseline in 2005 (Table 1), about one third of the cohort were less than 30 years old, slightly more than half were female, about 40% reported monthly income of less than 7000 Baht per month (175 US$ in 2005), 18% lived in Bangkok, and about half were urban residents. Injury deaths were more likely to affect males (73.7%, 15 vs 23 per 10000 person-years for transport vs non-transport injuries). Also notable, injury deaths were disproportionately frequent in the Southern region (20.6%, 12 vs 25 per 10000 person-years for transport vs non-transport injuries).

For social and health attributes (Table 2), a history of ever drink driving in the past year was more common among injury deaths (42.4% compared to 26.5% for other deaths or 25.4% for alive) and notably higher for transport than non-transport injuries (52.9% vs 35.0%). Cohort members who died from non-injury related causes were twice as likely to have reported poor self-assessed health at baseline and three times as likely to have reported metabolic and cardiovascular chronic conditions. Cohort members who died from injury reported higher rates of ever having been doctor-diagnosed depression (6.9% compared to 3.4% among non-deaths). As well, a history of depression was much more frequent for those who died from non-transport injuries than for transport injuries (34 vs 13 per 10000 person-years). At baseline in 2005, about 20% of cohort members overall reported injury at least once in the past year compared to 33.3% of cohort participants who died from transport injury.

For peer review only

In addition to analysing by injury types, we also tabulated the death rates according to the International Classification of Diseases (Table 3). Within transport injury mortality, rates per 10000 person-years for motorcycle riders and car occupants were 1.6 and 1.7, respectively. There were also 4.5 per 10000 person-years who died in unspecified motor vehicles. Among non-transport injury deaths, the rate per 10000 person-years of assault by firearm discharge was 2.5 with an additional 1.5 deaths from firearm discharge with undetermined intent. Deaths from drowning and submersion were 1.3 per 10000 person-years. Intentional self-harm deaths included self-poisoning and hanging-strangulation-suffocation with death rates of 0.3 and 0.8 per 10000 person-years, respectively.

To examine predictors of injury deaths (Table 4), we used multinomial logistic regression with three outcome categories: alive (reference), non-injury deaths, and injury deaths (study outcome). Highlighted in bold were results that were statistically significant at p<0.05. In the first column of odds ratios, the results are adjusted for age and sex; in the second column the odds ratios are adjusted for all covariates. All odds ratios compare the odds of injury death to the odds of staying alive.

We first calculate age-sex adjusted odds ratios for each potential exposure and for covariates. In the age-sex adjusted mode, being younger than 30 years, being male, residing in the South, currently smoking, drink driving in the past year, ever having been diagnosed for depression, having low social support, injury incidence in the year preceding baseline, and reported road injury in 2005, all were associated with injury mortality.

We then proceed to multivariate analysis (Table 4). After mutually adjusting for all tabulated covariates, statistically significant predictors of injury mortality were being male (Adjusted Odds Ratio, AOR 3.87), residing in the Southern areas (AOR 1.71), being a current smoker (AOR 1.56), history of drink driving (AOR 1.49) and ever having been diagnosed for depression (AOR 1.91). Other covariates such as being young, having low social support, and reporting road injury in the past year at baseline had predictive AORs ranging from 1.4 to 1.6, but these substantive estimates were not statistically significant for overall injury. Further investigation into types of injury (data not shown) revealed that, younger age was a strong predictor for transport injury deaths (AOR 4.12, 95% 1.03-16.5) and low social support for non-transport injury deaths (AOR 1.64, 95% 1.04-2.59).

For peer review only

In marked contrast to the injury deaths, cohort members who died from other causes had different sets of statistically significant risks at 2005 baseline which included older age, residing in Bangkok, reporting poor self-assessed health, and having metabolic or cardiovascular chronic conditions (data not shown). The only risk factor that non-injury deaths have in common with injury deaths was being a current smoker.

Discussion

This study is embedded in an overarching investigation of health-risk transition in Thailand, where similar to middle-income Southeast Asian countries; injury has been and still is a major population health burden. We make use of our large cohort of adults to investigate risk factors associated with injury mortality, linking deaths over five years (2005-2010) to individual characteristics and social and health attributes recorded at baseline (2005). The results revealed that injuries constituted almost one third of all deaths in the cohort, and some 40% of those were from transport and nearly 60% were non-transport injuries. Connecting to baseline information provided five years earlier, we identified epidemiologically and statistically significant predictors of injury mortality. These predictors included certain geo-demographic characteristics (male sex, Southern residents), health risk behaviours (smoking and history of drink driving), and adverse health diagnoses (depression).

Our injury mortality findings provide further information adding to our previous knowledge on risk factors associated with injury morbidity in the cohort, especially being male, having a history of depression, and drink driving.[18-20] The effect of smoking on injury was also shown in other studies after controlling for covariates including alcohol drinking. Plausible explanations include accidental fire hazard and distraction or inattention for road traffic hazards.[25, 26] Non-transport injury deaths presented here reflected the current political situation with violence in the Southern part of Thailand since 2004.[28, 29] For covariates with substantial (but not statistically significant) point estimates of overall injury death (young age, low social support, history of road injury), AORs ranged from 1.4-1.6.

Our results support other published international information on risk factors related to injury mortality, showing young males and drunk drivers at high risk of road crash death.[30, 31] A systematic review on alcohol consumption and collision risk concluded that there is no safe

For peer review only

level of drinking and even less than two drinks per occasion can almost double the odds of most types of injury.[30]

Our study also found social capital to be protective against injury mortality, supporting previous research.[32-34] Indeed, we found low social support to be a predictor of non-transport injury mortality. But social support did not relate to transport injury mortality. Despite the relatively small number of deaths in our study, we found that low social support was particularly associated with intentional self-harm and assault but these associations were not statistically significant.

The advantage of our study is its large participation by Thai adults who completed a comprehensive 20-page baseline questionnaire in 2005, reporting on a wide array of social and health characteristics. This provided a platform for investigation of causal pathways and elimination of many confounders. While our cohort members share similar distribution of sex, modest income, and geographical residence with the general Thai population. However, all cohort members completed high school education which facilitated their ability to respond to our detailed questionnaire. We also noted that a few (<5%) cohort members were 'missing' data for variables used in various models but this problem was not numerically significant. We acknowledge that our study captured the 5-year mortality rate after the baseline questionnaire in 2005 and the number of deaths was relatively small. However, the citizen IDs provided by cohort members at the baseline can be used to monitor patterns of mortality into the future and eventually a full account of cohort mortality will be possible.

Our research contributes to limited longitudinal evidence linking risk factors to injury death and is one of the first studies in middle-income Southeast Asia. We have achieved our aim of identifying vulnerable population subgroups at risk of injury deaths. As well, we complemented routinely collected administration death registration with a longitudinal health assessment providing information on individual, social, and health history factors. This information is invaluable in yielding insight into certain risk traits such as being a young male, reporting and having a past medical diagnosis of depression, which could inform injury prevention policies and strategies suitable for transitional countries with limited resources and changing patterns of mortality.

For peer review only

Contributors

VY conceptualized, analysed and drafted the manuscript. JB-G, RM, CB provided inputs to improve the draft. SS and AS designed and executed the Thai Cohort Study. AS provided comments and guidance on various revisions. All authors read and approved the final manuscript.

Author footnote

The Thai Cohort Study Team

Thailand: Jaruwan Chokhanapitak, Suttanit Hounthasarn, Suwanee Khamman, Daoruang Pandee, Suttinan Pangsap, Tippawan Prapamontol, Janya Puengson, Sam-ang Seubsman, Boonchai Somboonsook, Nintita Sripaiboonkij, Pathumvadee Somsamai, Duangkae Vilainerun, Wanee Wimonwattanaphan, Tewarit Somkotra, Benjawan Tawatsupa, Wimalin Rimpeekool.

Australia: Chris Bain, Emily Banks, Cathy Banwell, Bruce Caldwell, Gordon Carmichael, Tarie Dellora, Jane Dixon, Sharon Friel, David Harley, Matthew Kelly, Tord Kjellstrom, Lynette Lim, Anthony McMichael, Tanya Mark, Penny Haora, Adrian Sleigh, Lyndall Strazdins, Susan Jordan, Roderick McClure, Janneke Berecki-Gisolf, Vasoontara Yiengprugsawan.

Acknowledgments

We thank the staff at Sukhothai Thammathirat Open University (STOU) who assisted with student contact and the STOU students who are participating in the cohort study. We also thank Dr Bandit Thinkamrop and his team from Khon Kaen University for support on initial data processing. As well we would like thank the Thai Ministry of Interior and Ministry of Public Health for mortality investigation and cause of death data linkage. Matthew Kelly and Peter Sbirakos provided language editorial support.

Funding

This study was supported by the International Collaborative Research Grants Scheme with joint grants from the Wellcome Trust UK (GR071587MA) and the Australian National Health and Medical Research Council (268055), and as a global health grant from the NHMRC (585426).

Ethical approval

Informed written consent was obtained from all participants. All students were advised that they could withdraw, or not participate, without any effect on their academic progress. The questionnaires never sought sensitive personal information and no biological samples were taken. Ethics approval was obtained from Sukhothai Thammathirat Open University Research and Development Institute (protocol 0522/10) and the Australian National University Human Research Ethics Committee (protocols 2004/344 and 2009/570).

Competing interests

None declared.

Data sharing statement

Data are available through a data access agreement which includes guarantees regarding ethical conduct and scientific quality of any proposed analyses and publications. Anyone wanting access should contact Professor Adrian Sleigh or Associate Professor Sam-ang Seubsman (Thai Cohort Study Principal Investigators).

For peer review only

Table 1 Distribution of mortality by baseline social and geo-demographic attributes, Thai Cohort Study

Cohort attributes	Vital status by attributes %					Incidence per 10000 person-years	
	Alive (86457)	Other deaths (376)	Injury deaths (204)	Injury deaths (204)		Transport (84)	Non-transport (120)
				Transport (84)	Non-transport (120)		
**Age groups in years:**							
18-29	31.4	27.7	27.9	20.2	25.7	6	15
30-39	53.6	29.5	59.3	69.1	51.4	12	14
40-49	12.5	25.8	9.3	6.0	17.1	5	13
≥50	2.4	17.0	3.4	4.8	5.7	19	14
**Sex:**							
Males	45.2	65.6	73.7	69.4	76.7	15	23
**Marital status:**							
Married	38.8	50.1	35.0	30.9	37.7	8	14
Not married	56.8	41.9	60.1	64.3	57.1	11	14
Divorced/widowed	4.4	8.0	4.9	4.8	5.0	11	16
**Personal monthly income:**							
≤3000 Baht	11.0	14.9	14.6	11.9	16.5	10	20
3001-7000	30.9	22.4	33.2	39.3	28.7	13	13
7001-10000	23.3	16.6	23.6	26.2	21.7	11	13
10001-20000	24.2	28.7	20.6	16.7	23.5	7	13
>20000	10.5	17.4	8.0	6.0	9.6	6	12
**Regions:**							
Central/East	30.7	30.7	24.9	23.8	24.2	8	11
Bangkok	17.2	18.7	10.7	9.4	11.7	5	9
North	18.2	22.7	21.0	23.5	19.2	13	15
Northeast	20.9	21.6	23.4	25.9	21.7	12	14
South	13.0	12.3	20.6	16.7	23.3	12	25
**Lifecourse residence:**							
Rural-rural	43.3	42.3	46.6	38.1	52.5	8	17
Rural-urban	31.5	25.0	16.7	28.6	30.8	9	13
Urban-rural	4.2	6.9	4.4	7.1	2.5	16	8
Urban-urban	19.7	22.3	16.7	22.6	12.5	11	9

For peer review only - http://bmjopen.bmj.com/site/about/guidelines.xhtml

13

Table 2 Mortality by baseline health-risk behaviours and states, social attributes, and history of injury, Thai Cohort Study

Social and health attributes	Vital status by attributes %					Incidence per 10000 person-years	
	Alive (86457)	Other deaths (376)	Injury deaths (204)	Injury deaths (204) Transport (84)	Non-transport (120)	Transport (84)	Non-transport (120)
**Health-risk attributes**							
**Smoking:**							
Never	72.3	51.1	57.5	66.3	51.3	9	10
Current	10.0	19.8	24.0	21.7	25.6	21	35
Former	15.8	26.1	15.5	9.6	19.7	6	17
**Alcohol drinking:**							
Never	26.5	22.9	19.8	21.4	18.6	8	10
Occasional	59.8	49.5	60.9	61.9	60.2	10	14
Regular	4.8	9.7	7.9	9.5	6.8	19	19
Stop	8.9	18.0	11.4	7.1	14.4	8	22
**Ever drink driving in past year:**							
Yes	25.4	26.5	42.4	52.9	35.0	20	19
Do not usually drive	8.8	8.2	8.8	9.4	8.3	10	13
**Health and social attributes**							
**Self-assessed health:**							
Poor or very poor	4.6	8.8	3.9	2.4	5.0	7	16
**Chronic conditions:**							
Yes	12.5	29.3	10.8	13.1	9.2	10	13
**Doctor diagnosed depression:**							
Yes	3.4	5.9	6.9	4.8	8.3	13	34
**Social capital:**							
Low trust	38.2	36.9	34.5	35.4	33.9	9	12
Low support	25.5	33.2	20.1	22.6	18.3	9	20
Low interaction	23.3	25.5	28.9	22.6	33.3	9	10
**Injury reported in 2005**							
**Number of injuries:**							
At least once	20.2	29.5	27.9	33.3	24.2	16	16
**Location of injury:**							
Home	5.3	7.1	5.4	5.9	5.0	10	14
Road	5.9	4.8	11.7	16.5	8.3	25	20
Work	3.9	6.4	5.4	9.5	2.5	11	13

For peer review only

Table 3 Injury mortality by International Classification of Diseases (ICD-10), Thai Cohort Study

Types of injury deaths	Number of deaths	Rate per 10000 person-years
**Transport injuries**		
V01-V09 Pedestrian	2	0.2
V20-V29 Motorcycle rider	14	1.6
V40-V49 Car occupant	15	1.7
V50-59 Occupant of pick-up truck or van	3	1.0
V80-V89 Other land transport accident	9	0.1
V89.2 Person injured in unspecified motor vehicle	39	4.5
V90-V94 Water transport	1	0.1
V95-V97 Air and space transport	1	0.1
**Non-transport injuries**		
W00-W19 Falls	2	0.3
W65-W74 Drowning and submersion	10	1.3
W75-W84 Other threats to breathing	1	0.1
W87 Exposure to electric current	2	0.3
X00-X09 Exposure to smoke, fire and flames	2	0.3
X33 Victim of lighting	1	0.1
X38 Victim of flood	1	0.1
X58-X59 Exposure to other unspecified factors	16	2.1
X60-X84 Intentional self-harm		
X65 Intentional self-poisoning	3	0.3
X70 Intentional self-harm by hanging, strangulation and suffocation	7	0.8
X85-Y09 Assault		
X95 Assault by unspecified firearm discharge	22	2.5
X99 Assault by sharp object	3	0.3
Y99 Assault by other unspecified means	5	0.6
Y10-Y34 Event of undetermined intent		
Y18 Poisoning by and exposure to pesticides	1	0.1
Y20 Hanging, strangulation and suffocation	3	0.3
Y22-Y24 Firearm discharge, undetermined intent	13	1.5
Y25 Contact with explosive material	1	0.1
Y28-Y29 Contact with sharp of blunt object	4	0.5
Y34 Unspecified event, undetermined intent	23	2.6

For peer review only

Table 4 Age-sex adjusted and multivariate predictors of injury mortality, Thai Cohort Study

2005 baseline covariates	Multinomial* Adjusted Odds Ratios [95% CI]			
	Age-sex adjusted		Multivariate**	
	Alive	Injury death	Alive	Injury death
**Geo-demographic covariates**				
**Age groups in years:** 20-29		**1.45 [1.06-1.99]**		1.51 [0.99-2.32]
30-39	*Ref*		*Ref*	
40-49		0.76 [0.45-1.28]		0.99 [0.54-1.83]
≥50		1.29 [0.59-2.84]		1.65 [0.61-4.43]
**Sex** *(female):*	*Ref*		*Ref*	
Male		**3.69 [2.69-5.07]**		**3.87[2.39-6.26]**
**Marital status** *(married):*	*Ref*		*Ref*	
Not married		1.09 [0.77-1.56]		1.11 [0.73-1.69]
Divorced/widowed		1.55 [0.80-3.02]		1.47 [0.69-3.14]
**Personal monthly income:**				
≤3000Baht		1.24 [0.80-1.93]		1.10 [0.48-1.53]
3001-7000		1.25 [0.89-1.75]		1.15 [0.78-1.69]
7001-20000		0.81 [0.46-1.43]		0.83 [0.43-1.61]
>20000	*Ref*		*Ref*	
**Regions** (*Central/East*):	*Ref*		*Ref*	
Bangkok		0.85 [0.51-1.41]		0.86 [0.48-1.53]
North		1.38 [0.92-2.08]		1.27 [0.80-2.01]
Northeast		1.29 [0.87-1.93]		0.98 [0.61-1.56]
South		**1.96 [1.30-2.97]**		**1.71 [1.05-2.79]**
**Lifecourse residence** *(rural-rural):*	*Ref*		*Ref*	
Urban-rural		0.91 [0.66-1.27]		0.97 [0.65-1.44]
Rural-urban		1.03 [0.52-2.04]		1.32 [0.73-1.44]
Urban-urban		0.87 [0.59-1.29]		1.12 [0.71-1.77]
**Health and social covariates**				
**Smoking** *(never):*	*Ref*		*Ref*	
Current		**1.70 [1.07-2.05]**		**1.56 [1.03-2.37]**
Former		0.77 [0.45-1.32]		0.74 [0.47-1.18]
**Alcohol drinking** *(never):*	*Ref*		*Ref*	
Occasional		0.87 [0.60-1.27]		0.40 [0.12-1.30]
Regular		1.06 [0.58-1.95]		0.38 [0.10-1.40]
Stop		1.08 [0.64-1.84]		0.63 [0.18-2.15]
**Drink driving past year** *(never):*	*Ref*		*Ref*	
Yes		**1.50 [1.07-2.12]**		**1.49 [1.01-2.20]**
**Self-assessed health** *(good):*	*Ref*		*Ref*	
Poor or very poor		1.09 [0.58-2.07]		0.75 [0.32-1.71]
**Depression** *(no):*	*Ref*		*Ref*	
Yes		**2.15 [1.25-3.71]**		**1.91 [1.00-3.69]**
**Chronic illness** *(no):*	*Ref*		*Ref*	
Yes		0.80 [0.50-1.2]		0.84 [0.50-1.44]
**Social capital:**				
Low social trust (*ref: not low*)		084 [0.60-1.19]		0.90 [0.76-1.07]
Low social support (*ref*: not low)		1.29 [0.58-2.84]		1.37 [0.96-1.96]
Low social interaction (*ref*: not low)		0.86 [0.64-1.15]		0.77 [0.50-1.20]
**Injury reported in 2005**				
**Injuries in the past year** *(no):*	*Ref*		*Ref*	
At least once		**1.41 [1.04-1.92]**		1.12 [0.70-1.82]
**Location of injury:**				
Home (*ref: no*)		1.13 [0.62-2.08]		1.19 [0.56-2.55]
Road (*ref: no*)		**1.81 [1.17-2.79]**		1.58 [0.87-2.84]
Work (*ref: no*)		1.22 [0.66-2.25]		1.15 [0.54-2.44]

*multinomial logistic regression compares the odds of injury deaths to the odds of remaining alive by predictor covariate category values, after adjusting for age-sex or other covariates.

**mutually adjusted for all predictor covariates presented in this table

For peer review only

References

1. Stevenson M. Measuring the global burden of road traffic injury: implications for low-income and middle-income countries. Inj Prev. 2009; 15:1-2.
2. Lim SS, Vos T, Flaxman AD, Danaei G, Shibuya K, Adair-Rohani H, et al. A comparative risk assessment of burden of disease and injury attributable to 67 risk factors and risk factor clusters in 21 regions, 1990-2010: a systematic analysis for the Global Burden of Disease Study 2010. Lancet. 2012; 380:2224-60.
3. WHO. Global status report on road safety 2013: supporting a decade of action. Geneva: World Health Organization; 2013.
4. WHO. World report on road traffic injury prevention. Geneva: World Health Organization; 2004.
5. Chandran A, Hyder AA, Peek-Asa C. The global burden of unintentional injuries and an agenda for progress. Epidemiol Rev. 2010; 32:110-20.
6. WHO. Global status report on violence prevention. Geneva: World Health Organization; 2014.
7. Bhalla K, Harrison J, Abraham J, Borse NN, Lyons R, Boufous S, et al. Data sources for improving estimates of the global burden of injuries: call for contributors. PLoS Med. 2009; 6 e1 doi: 10.1371/journal.pmed.1000001.
8. Bhalla K, Harrison JE, Fingerhut LA, Shahraz S, Abraham J, Yeh PH, et al. The global injury mortality data collection of the Global Burden of Disease Injury Expert Group: a publicly accessible research tool. Int J Inj Contr Saf Promot. 2011; 18:249-53.
9. Baker SP, Li G. Epidemiologic approaches to injury and violence. Epidemiol Rev. 2011; 34:1-3.
10. Mathers CD, Fat DM, Inoue M, Rao C, Lopez AD. Counting the dead and what they died from: an assessment of the global status of cause of death data. Bull World Health Organ. 2005; 83:171.
11. Ditsuwan V, Veerman LJ, Barendregt JJ, Bertram M, Vos T. The national burden of road traffic injuries in Thailand. Popul Health Metr. 2011; 9:2; doi: 10.1186/478-7954-9-2.
12. Chadbunchachai W, Suphanchaimaj W, Settasatien A, Jinwong T. Road traffic injuries in Thailand: current situation. J Med Assoc Thai. 2012; 95 Suppl 7:S274-81.
13. Choprapawon C, Porapakkham Y, Sablon O, Panjajaru R, Jhantharatat B. Thailand's national death registration reform: verifying the causes of death between July 1997 and December 1999. Asia Pac J Public Health. 2005; 17:110-6.
14. Tangcharoensathien V, Faramnuayphol P, Teokul W, Bundhamcharoen K, Wibulpholprasert S. A critical assessment of mortality statistics in Thailand: potential for improvements. Bull World Health Organ. 2006; 84:233-8.
15. Rao C, Porapakkham Y, Pattaraarchachai J, Polprasert W, Swampunyalert N, Lopez AD. Verifying causes of death in Thailand: rationale and methods for empirical investigation. Popul Health Metr. 2010; 8:11.
16. Porapakkham Y, Rao C, Pattaraarchachai J, Polprasert W, Vos T, Adair T, et al. Estimated causes of death in Thailand, 2005: implications for health policy. Popul Health Metr. 2010; 8:14.
17. Pattaraarchachai J, Rao C, Polprasert W, Porapakkham Y, Pao-In W, Singwerathum N, et al. Cause-specific mortality patterns among hospital deaths in Thailand: validating routine death certification. Popul Health Metr. 2010; 8:12.
18. Yiengprugsawan V, Stephan K, McClure R, Kelly M, Seubsman S, Bain C, et al. Risk factors for injury in a national cohort of 87,134 Thai adults. Public Health. 2012; 126:33-9.
19. Stephan K, Kelly M, McClure R, Seubsman SA, Yiengprugsawan V, Bain C, et al. Distribution of transport injury and related risk behaviours in a large national cohort of Thai adults. Accid Anal Prev. 2011; 43:1062-7.
20. Tawatsupa B, Yiengprugsawan V, Kjellstrom T, Berecki-Gisolf J, Seubsman SA, Sleigh A. Association between heat stress and occupational injury among Thai workers: findings of the Thai Cohort Study. Ind Health. 2013; 51:34-46.
21. Sleigh AC, Seubsman SA, Bain C. Cohort profile: The Thai Cohort of 87,134 Open University students. Int J Epidemiol. 2008; 37:266-72.
22. Seubsman SA, Yiengprugsawan V, Sleigh AC, the Thai Cohort Study T. A large national Thai Cohort Study of the Health-Risk Transition based on Sukhothai Thammathirat Open University students. ASEAN J Open Distance Learn. 2012; 4:58-69.
23. Prasartkul P, Vapattanawong P. The completeness of death registration in Thailand: evidence from demographic surveillance system of the kanchanaburi project. World Health Popul. 2006; 8:43-51.
24. WHO. International Statistical Classification of Diseases and Related Health Problems, 10th Revision. http://apps.who.int/classifications/icd10/browse/2010/en: World Health Organization.; 2011.
25. Leistikow BN, Martin DC, Jacobs J, Rocke DM, Noderer K. Smoking as a risk factor for accident death: a meta-analysis of cohort studies. Accid Anal Prev. 2000; 32:397-405.

For peer review only
For peer review only - http://bmjopen.bmj.com/site/about/guidelines.xhtml

26. Wen CP, Tsai SP, Cheng TY, Chan HT, Chung WS, Chen CJ. Excess injury mortality among smokers: a neglected tobacco hazard. Tobacco control. 2005; 14 Suppl 1:i28-32.

27. Patten SB, Williams JV, Lavorato DH, Eliasziw M. Major depression and injury risk. Can J Psychiatry. 2010; 55:313-8.

28. Bonura C. Indeterminate geographies of political violence in Southern Thailand. Alternatives. 2008; 33:383-412.

29. McCargo D. Rethinking Thailand's southern violence - Introduction. Crit Asian Stud. 2006; 38:3-10.

30. Taylor B, Irving HM, Kanteres F, Room R, Borges G, Cherpitel C, et al. The more you drink, the harder you fall: a systematic review and meta-analysis of how acute alcohol consumption and injury or collision risk increase together. Drug Alcohol Depend. 2010; 110:108-16.

31. Connor J, Norton R, Ameratunga S, Jackson R. The contribution of alcohol to serious car crash injuries. Epidemiology. 2004; 15:337-44.

32. Aida J, Kondo K, Hirai H, Subramanian SV, Murata C, Kondo N, et al. Assessing the association between all-cause mortality and multiple aspects of individual social capital among the older Japanese. BMC Public Health. 2011; 11:499.

33. Hyyppa MT, Maki J, Impivaara O, Aromaa A. Individual-level measures of social capital as predictors of all-cause and cardiovascular mortality: a population-based prospective study of men and women in Finland. Eur J Epidemiol. 2007; 22:589-97.

34. Muennig P, Cohen AK, Palmer A, Zhu W. The relationship between five different measures of structural social capital, medical examination outcomes, and mortality. Soc Sci Med. 2013; 85:18-26.

For peer review only

BMJ Open

Predictors of injury mortality: findings from a large national cohort in Thailand

Journal:	*BMJ Open*
Manuscript ID:	bmjopen-2013-004668.R2
Article Type:	Research
Date Submitted by the Author:	19-May-2014
Complete List of Authors:	Yiengprugsawan, Vasoontara; The Australian National University, National Centre for Epidemiology and Population Health<br>Berecki-Gisolf, Janneke; Monash Injury Research Institute,<br>Bain, Christopher; Queensland Institute of Medical Research, Genetics and Population Health Division<br>McClure, Roderick; Monash Injury Research Institute,<br>Seubsman, Sam-ang; Sukhothai Thammathirat Open University,<br>Sleigh, Adrian; The Australian National University, National Centre for Epidemiology and Population Health
**Primary Subject Heading**:	Epidemiology
Secondary Subject Heading:	Epidemiology, Global health, Public health
Keywords:	Epidemiology < THORACIC MEDICINE, Suicide & self-harm < PSYCHIATRY, PUBLIC HEALTH

SCHOLARONE™
Manuscripts

For peer review only

Predictors of injury mortality: findings from a large national cohort in Thailand

Vasoontara Yiengprugsawan[1§], Janneke Berecki-Gisolf[2], Christopher Bain[3], Roderick McClure[2], Sam-ang Seubsman[1,4], Adrian C Sleigh[1]

[1] National Centre for Epidemiology and Population Health, The Australian National University, Canberra, Australia
[2] Monash Injury Research Institute, Monash University, Melbourne, Australia
[3] Genetics and Population Health Division, Queensland Institute of Medical Research, Brisbane, Queensland, Australia
[4] School of Human Ecology, Sukhothai Thammathirat Open University, Nonthaburi, Thailand

[§] **Correspondence to:** Dr. Vasoontara Yiengprugsawan, The Australian National University, College of Medicine, Biology and Environment, Research School of Population Health, National Centre for Epidemiology and Population Health, Building 62, Mills Rd, Acton 2601, Canberra, Australia Tel: +61 2 6125 2378; Fax: +61 2 6125 0740
Email vasoontara.yieng@anu.edu.au; vasoontara.yieng@gmail.com

For peer review only

Predictors of injury mortality: findings from a large national cohort in Thailand

Abstract

Objective: to present predictors of injury mortality by types of injury and by pre-existing attributes or other individual exposures identified at baseline.

Design: 5-year prospective longitudinal study

Setting: Contemporary Thailand (2005-2010), a country undergoing epidemiological transition

Participants: Data derived from a research cohort of 87,037 distance-learning students enrolled at Sukhothai Thammathirat Open University residing nationwide.

Measures: Cohort members completed a comprehensive baseline mail-out questionnaire in 2005 reporting geo-demographic, behavioural, health, and injury data. These responses were matched with national death records using the Thai Citizen ID number. Age-sex adjusted multinomial logistic regression was used to calculate odds ratios linking exposure variables collected at baseline to injury deaths over the next five years.

Results: Statistically significant predictors of injury mortality were being male (Adjusted Odds Ratio 3.87, 95% Confidence Interval 2.39-6.26), residing in the Southern areas (AOR 1.71, 1.05-2.79), being a current smoker (1.56, 1.03-2.37), history of drink driving (AOR 1.49,1.01-2.20) and ever having been diagnosed for depression (AOR 1.91, 1.00-3.69). Other covariates such as being young, having low social support, and reporting road injury in the past year at baseline had moderately predictive AORs ranging from 1.4 to 1.6 but were not statistically significant.

Conclusions: We complemented national death registration with longitudinal data on individual, social, and health attributes. This information is invaluable in yielding insight into certain risk traits such as being a young male, history of drink driving, and history of depression. Such information could be used to inform injury prevention policies and strategies.

For peer review only

Article summary:

Strengths and limitations of this study:

- Injury is a population health burden in transitional low and middle-income Southeast Asia. We investigated a large national cohort of Thai adults for predictors of injury mortality including geo-demographic, social, and health attributes recorded at baseline.
- Injuries constituted almost one third of all deaths in the cohort, and some 40% of those were from transport and nearly 60% were non-transport injuries. These injury mortality observations add to our previous Thai work on injury morbidity, highlighting the overall risks, especially depression, male sex, and drink driving.
- The advantage of our study is its large size, longitudinal design, and comprehensive baseline information. This provides a platform for identification of risks, elimination of confounders, and exploration of causal pathways.
- This study captured the 5-year mortality rate in a generally young adult cohort. Thus there were relatively few deaths. Citizen IDs provided at baseline will enable us to monitor patterns of cohort mortality into the future.

For peer review only

Introduction

Injury remains a major public health challenge worldwide; causing one tenth of global mortality with a heavy burden in developing countries.[1, 2] According to the World Health Organization, at least 1.2 million people are killed from road crashes and an estimated 50 million are injured on roads worldwide each year.[3, 4] Violence and non-transport injuries also accounted for more than 1.3 million deaths and many suffered from serious physical and mental consequences.[5, 6]

Most national injury prevention policies have been introduced in high-income countries. Unfortunately, very few low and middle-income countries have been able to develop such policies due to lack of resources and limited availability of quality injury mortality data.[7, 8] In particular, many developing countries still face the challenges of accurately identifying causes of death from routinely collected national civil registration and vital statistics systems while other sources of data such as police reports and hospital records are never comprehensive and lead to underreporting bias if relied upon as a main source of injury mortality data. Reliable cause-of-death data are important because they enable monitoring of the epidemiological occurrence and public health effects at the population level.[9, 10]

Throughout middle-income Southeast Asia, including Thailand, injury continues to be one of the top ten causes of death.[11, 12] In past decades, Thailand has reformed administrative records to improve the coverage and quality of cause-specific mortality data.[13, 14] Eight years ago, Thailand began to study the ill-defined causes of death by using verbal autopsies and these revealed that besides a high proportion of transport-related deaths, a number of other deaths which were initially recorded as non-specific causes turned out to be suicides, assaults, and drowning.[15-17] These findings shed light on the importance of non-transport injuries in addition to the burden of transport injuries.

This study is based on a large national cohort in Thailand which has been followed to investigate health-risk transitions of Thai adults since 2005. The cohort database includes comprehensive information on individual characteristics, social demography, health behaviours and specific diseases, as well as history of injuries. Our previous research based on this cohort has examined risk factors associated with injury morbidity.[18-20] Now successful mortality data linkage

For peer review

through the Thai Ministry of Interior and Ministry of Public Health allows us to analyse injury-related deaths among the cohort over the first five years (2005-10).

Informed by our earlier research on injury morbidity, and by related published information, this study has investigated injury in more depth using mortality as the outcome. Our study linked cohort outcomes (survival, non-injury death, injury death) to an array of relevant exposures recorded at baseline including geo-demographic attributes, social covariates, health and psychological states, and health-risk behaviours. This analysis is both prospective and cohort-based and fills an important gap regarding our knowledge of injury risks as an emerging public health problem in a middle-income Asian country going through the health-risk transition.

Methods

Study population and data collection

This analysis is part of the overarching Thai Cohort Study (TCS), an ongoing epidemiological investigation of changing patterns of health risks and outcomes. Data derive from a research cohort of 87,037 distance-learning adult students enrolled at Sukhothai Thammathirat Open University, who resided all over Thailand and completed the baseline comprehensive mail-out health questionnaire in 2005 (response rate 44%). The cohort participants recapitulated well the distance learning student body at STOU and share certain geo-demographic attributes with the general Thai population (mean age was 29 years in 2005, slightly more than half were females, half resided in urban areas).[21, 22] The baseline questionnaire gathered data on a wide range of topics including age, sex, income, marital status, health status, doctor-diagnosed diseases, health-risk behaviors including smoking and drinking, social capital, and history of injury.

Mortality data

The completeness of death registration in Thailand was 86% from 1950-2000[10], but over the last decade coverage improved to 95%.[23] A powerful feature of our study is that all cohort members have provided their Thai Citizen ID number enabling detection and analysis of deaths into the future. These confidential ID numbers were safeguarded and stored at STOU in a secure office on the main campus with 24-hour guards on patrol. The working files of these data were de-identified and no individual information will be released or displayed in any format. To detect

For peer review only

deaths, the Bangkok TCS team periodically matched the cohort against national death records from the Ministry of Interior using the Citizen ID number. At a later stage the Thai Ministry of Public Health expanded these death records by adding the standard International Classification of Diseases (ICD-10)[24] to identify causes of death.

Up until March 2010, there were a total of 580 deaths among the Thai Cohort Study participants. According to the ICD-10 codes, there were 376 deaths from non-injury causes including ill-defined causes of death. For the purpose of this study, these will not be broken down and will be designated as 'other deaths'. For our injury-focused death analysis, there were 204 deaths from external causes, including 84 deaths from transport accidents. Among the 120 non-transport injury deaths, there were 35 deaths from miscellaneous external causes, 10 deaths from intentional self-harm, 30 deaths from assault, and 45 deaths from 'unspecified events of undetermined intent'.

Exposures and confounders

In our analysis, exposures of interest and potential confounders from the 2005 baseline questionnaire included the following geo-demographic variables: age (4 categories), sex, marital status (married, not married, divorced/ widowed), personal monthly income (≤3000 Baht, 3001-7000, 7001-10000, 10001-20000, >20000), regions (Central/East, Bangkok, North, Northeast, South), and lifecourse urbanisation (residence at age 12 years old and at baseline: rural-rural, rural-urban, urban-rural, urban-urban). As well, a history of injury in the past year was reported at 2005 baseline, including the frequency and location of injuries reported.

Also analysed as exposures of interest were certain social covariates, several health states, and important health-risk behaviours. These behaviours included smoking and alcohol drinking which have been shown to be independently associated with injury.[25, 26] Smoking status includes never, current, and former and alcohol status includes never, occasional, regular, and former. In addition, at baseline cohort members were asked "in the last year have you driven a motor vehicle after consuming 3 or more glasses of alcohol" (ie drink driving). Other health-related attributes included self-assessed health and chronic metabolic or cardiovascular disorders (e.g. diabetes, hypertension). A history of doctor-diagnosed depression has previously been shown to be an injury risk[18, 27] and is also included in the model. Social capital was dichotomoised for analyses (low or not low) in three domains: trust (whether people can be trusted), support (from family, friends, colleagues), and interaction (with family, friends, neighbours).

For peer review only

Data processing and statistical analysis

Questionnaire responses were digitised by optical scanning and subsequently edited using Thai Scandevet, SQL and SPSS software. For analysis we used Stata version 12. Individuals with missing data for given analyses were excluded (<5%), so totals vary slightly according to available information. We described the distribution of deaths by demographic, social, and health covariates, by cause of death, and by types of injury (transport and non-transport injuries). In addition, we presented death rates per 10,000 person-years of exposure. We then used age-sex adjusted multinomial logistic regression linking the above covariates to three possible outcomes: alive (reference), deaths from other causes, and deaths from injury (study outcome). The final analytical model mutually adjusted for all covariates and reported Adjusted Odds Ratios (AOR) and 95% Confidence Intervals.

Results

Among Thai cohort members at baseline in 2005 (Table 1), about one third of the cohort were less than 30 years old, slightly more than half were female, about 40% reported monthly income of less than 7000 Baht per month (175 US$ in 2005), 18% lived in Bangkok, and about half were urban residents. Injury deaths were more likely to affect males (73.7%, 15 vs 23 per 10000 person-years for transport vs non-transport injuries). Also notable, injury deaths were disproportionately frequent in the Southern region (20.6%, 12 vs 25 per 10000 person-years for transport vs non-transport injuries).

For social and health attributes (Table 2), a history of ever drink driving in the past year was more common among injury deaths (42.4% compared to 26.5% for other deaths or 25.4% for alive) and notably higher for transport than non-transport injuries (52.9% vs 35.0%). Cohort members who died from non-injury related causes were twice as likely to have reported poor self-assessed health at baseline and three times as likely to have reported metabolic and cardiovascular chronic conditions. Cohort members who died from injury reported higher rates of ever having been doctor-diagnosed depression (6.9% compared to 3.4% among non-deaths). As well, a history of depression was much more frequent for those who died from non-transport injuries than for transport injuries (34 vs 13 per 10000 person-years). At baseline in 2005, about 20% of cohort members overall reported injury at least once in the past year compared to 33.3% of cohort participants who died from transport injury.

For peer review only

In addition to analysing by injury types, we also tabulated the death rates according to the International Classification of Diseases (Table 3). Within transport injury mortality, rates per 10000 person-years for motorcycle riders and car occupants were 1.6 and 1.7, respectively. There were also 4.5 per 10000 person-years who died in unspecified motor vehicles. Among non-transport injury deaths, the rate per 10000 person-years of assault by firearm discharge was 2.5 with an additional 1.5 deaths from firearm discharge with undetermined intent. Deaths from drowning and submersion were 1.3 per 10000 person-years. Intentional self-harm deaths included self-poisoning and hanging-strangulation-suffocation with death rates of 0.3 and 0.8 per 10000 person-years, respectively.

To examine predictors of injury deaths (Table 4), we used multinomial logistic regression with three outcome categories: alive (reference), non-injury deaths, and injury deaths (study outcome). Highlighted in bold were results that were statistically significant at p<0.05. In the first column of odds ratios, the results are adjusted for age and sex; in the second column the odds ratios are adjusted for all covariates. All odds ratios compare the odds of injury death to the odds of staying alive.

We first calculate age-sex adjusted odds ratios for each potential exposure and for covariates. In the age-sex adjusted mode, being younger than 30 years, being male, residing in the South, currently smoking, drink driving in the past year, ever having been diagnosed for depression, injury incidence in the year preceding baseline, and reported road injury in 2005, all were associated with injury mortality.

We then proceed to multivariate analysis (Table 4). After mutually adjusting for all tabulated covariates, statistically significant predictors of injury mortality were being male (Adjusted Odds Ratio, AOR 3.87), residing in the Southern areas (AOR 1.71), being a current smoker (AOR 1.56), history of drink driving (AOR 1.49) and ever having been diagnosed for depression (AOR 1.91). Other covariates such as being young, having low social support, and reporting road injury in the past year at baseline had predictive AORs ranging from 1.4 to 1.6, but these substantive estimates were not statistically significant for overall injury. Further investigation into types of injury (data not shown) revealed that, younger age was a strong predictor for transport injury deaths (AOR 4.12, 95% 1.03-16.5) and low social support for non-transport injury deaths (AOR 1.64, 95% 1.04-2.59).

For peer review only

In marked contrast to the injury deaths, cohort members who died from other causes had different sets of statistically significant risks at 2005 baseline which included older age, residing in Bangkok, reporting poor self-assessed health, and having metabolic or cardiovascular chronic conditions (data not shown). The only risk factor that non-injury deaths have in common with injury deaths was being a current smoker.

Discussion

This study is embedded in an overarching investigation of health-risk transition in Thailand, where similar to middle-income Southeast Asian countries; injury has been and still is a major population health burden. We make use of our large cohort of adults to investigate risk factors associated with injury mortality, linking deaths over five years (2005-2010) to individual characteristics and social and health attributes recorded at baseline (2005). The results revealed that injuries constituted almost one third of all deaths in the cohort, and some 40% of those were from transport and nearly 60% were non-transport injuries. Connecting to baseline information provided five years earlier, we identified epidemiologically and statistically significant predictors of injury mortality. These predictors included certain geo-demographic characteristics (male sex, Southern residents), health risk behaviours (smoking and history of drink driving), and adverse health diagnoses (depression).

Our injury mortality findings provide further information adding to our previous knowledge on risk factors associated with injury morbidity in the cohort, especially being male, having a history of depression, and drink driving.[18-20] The effect of smoking on injury was also shown in other studies after controlling for covariates including alcohol drinking. Plausible explanations include accidental fire hazard and distraction or inattention for road traffic hazards.[25, 26] Non-transport injury deaths presented here reflected the current political situation with violence in the Southern part of Thailand since 2004.[28, 29] For covariates with substantial (but not statistically significant) point estimates of overall injury death (young age, low social support, history of road injury), AORs ranged from 1.4-1.6.

Our results support other published international information on risk factors related to injury mortality, showing young males and drunk drivers at high risk of road crash death.[30, 31] A systematic review on alcohol consumption and collision risk concluded that there is no safe

For peer review only

level of drinking and even less than two drinks per occasion can almost double the odds of most types of injury.[30]

Our study also found social capital to be protective against injury mortality, supporting previous research.[32-34] Indeed, we found low social support to be a predictor of non-transport injury mortality. But social support did not relate to transport injury mortality. Despite the relatively small number of deaths in our study, we found that low social support was particularly associated with intentional self-harm and assault but these associations were not statistically significant.

The advantage of our study is its large participation by Thai adults who completed a comprehensive 20-page baseline questionnaire in 2005, reporting on a wide array of social and health characteristics. This provided a platform for investigation of causal pathways and elimination of many confounders. While our cohort members share similar distribution of sex, modest income, and geographical residence with the general Thai population. However, all cohort members completed high school education which facilitated their ability to respond to our detailed questionnaire. We also noted that a few (<5%) cohort members were 'missing' data for variables used in various models but this problem was not numerically significant. We acknowledge that our study captured the 5-year mortality rate after the baseline questionnaire in 2005 and the number of deaths was relatively small. However, the citizen IDs provided by cohort members at the baseline can be used to monitor patterns of mortality into the future and eventually a full account of cohort mortality will be possible.

Our research contributes to limited longitudinal evidence linking risk factors to injury death and is one of the first studies in middle-income Southeast Asia. We have achieved our aim of identifying vulnerable population subgroups at risk of injury deaths. As well, we complemented routinely collected administration death registration with a longitudinal health assessment providing information on individual, social, and health history factors. This information is invaluable in yielding insight into certain risk traits such as being a young male, reporting and having a past medical diagnosis of depression, which could inform injury prevention policies and strategies suitable for transitional countries with limited resources and changing patterns of mortality.

For peer review only

Contributors

VY conceptualized, analysed and drafted the manuscript. JB-G, CB, RM provided inputs to improve the draft. SS and AS designed and executed the Thai Cohort Study. AS provided comments and guidance on revisions. All authors read and approved the final manuscript.

Author footnote

The Thai Cohort Study Team

Thailand: Jaruwan Chokhanapitak, Suttanit Hounthasarn, Suwanee Khamman, Daoruang Pandee, Suttinan Pangsap, Tippawan Prapamontol, Janya Puengson, Sam-ang Seubsman, Boonchai Somboonsook, Nintita Sripaiboonkij, Pathumvadee Somsamai, Duangkae Vilainerun, Wanee Wimonwattanaphan, Tewarit Somkotra, Benjawan Tawatsupa, Wimalin Rimpeekool.

Australia: Chris Bain, Emily Banks, Cathy Banwell, Bruce Caldwell, Gordon Carmichael, Tarie Dellora, Jane Dixon, Sharon Friel, David Harley, Matthew Kelly, Tord Kjellstrom, Lynette Lim, Anthony McMichael, Tanya Mark, Penny Haora, Adrian Sleigh, Lyndall Strazdins, Susan Jordan, Roderick McClure, Janneke Berecki-Gisolf, Vasoontara Yiengprugsawan.

Acknowledgments

We thank the staff at Sukhothai Thammathirat Open University (STOU) who assisted with student contact and the STOU students who are participating in the cohort study. We also thank Dr Bandit Thinkamrop and his team from Khon Kaen University for support on initial data processing. As well we would like thank the Thai Ministry of Interior and Ministry of Public Health for mortality investigation and cause of death data linkage. Matthew Kelly and Peter Sbirakos provided language editorial support.

Funding

This study was supported by the International Collaborative Research Grants Scheme with joint grants from the Wellcome Trust UK (GR071587MA) and the Australian National Health and Medical Research Council (268055), and as a global health grant from the NHMRC (585426).

Ethical approval

Informed written consent was obtained from all participants. All students were advised that they could withdraw, or not participate, without any effect on their academic progress. The questionnaires never sought sensitive personal information and no biological samples were taken. Ethics approval was obtained from Sukhothai Thammathirat Open University Research and Development Institute (protocol 0522/10) and the Australian National University Human Research Ethics Committee (protocols 2004/344 and 2009/570).

Competing interests

None declared.

Data sharing statement

Data are available through a data access agreement which includes guarantees regarding ethical conduct and scientific quality of any proposed analyses and publications. Anyone wanting access should contact Professor Adrian Sleigh or Associate Professor Sam-ang Seubsman (Thai Cohort Study Principal Investigators).

For peer review only

Table 1 Distribution of mortality by baseline social and geo-demographic attributes, Thai Cohort Study

Cohort attributes	Vital status by attributes %					Incidence per 10000 person-years	
	Alive (86457)	Other deaths (376)	Injury deaths (204)	Injury deaths (204)		Transport (84)	Non-transport (120)
				Transport (84)	Non-transport (120)		
**Age groups in years:**							
18-29	31.4	27.7	27.9	20.2	25.7	6	15
30-39	53.6	29.5	59.3	69.1	51.4	12	14
40-49	12.5	25.8	9.3	6.0	17.1	5	13
≥50	2.4	17.0	3.4	4.8	5.7	19	14
**Sex:**							
Males	45.2	65.6	73.7	69.4	76.7	15	23
**Marital status:**							
Married	38.8	50.1	35.0	30.9	37.7	8	14
Not married	56.8	41.9	60.1	64.3	57.1	11	14
Divorced/widowed	4.4	8.0	4.9	4.8	5.0	11	16
**Personal monthly income:**							
≤3000 Baht	11.0	14.9	14.6	11.9	16.5	10	20
3001-7000	30.9	22.4	33.2	39.3	28.7	13	13
7001-10000	23.3	16.6	23.6	26.2	21.7	11	13
10001-20000	24.2	28.7	20.6	16.7	23.5	7	13
>20000	10.5	17.4	8.0	6.0	9.6	6	12
**Regions:**							
Central/East	30.7	30.7	24.9	23.8	24.2	8	11
Bangkok	17.2	18.7	10.7	9.4	11.7	5	9
North	18.2	22.7	21.0	23.5	19.2	13	15
Northeast	20.9	21.6	23.4	25.9	21.7	12	14
South	13.0	12.3	20.6	16.7	23.3	12	25
**Lifecourse residence:**							
Rural-rural	43.3	42.3	46.6	38.1	52.5	8	17
Rural-urban	31.5	25.0	16.7	28.6	30.8	9	13
Urban-rural	4.2	6.9	4.4	7.1	2.5	16	8
Urban-urban	19.7	22.3	16.7	22.6	12.5	11	9

For peer review only

Table 2 Mortality by baseline health-risk behaviours and states, social attributes, and history of injury, Thai Cohort Study

Social and health attributes	Vital status by attributes %					Incidence per 10000 person-years	
	Alive (86457)	Other deaths (376)	Injury deaths (204)	Injury deaths (204)		Transport (84)	Non-transport (120)
				Transport (84)	Non-transport (120)		
**Health-risk attributes**							
**Smoking:**							
Never	72.3	51.1	57.5	66.3	51.3	9	10
Current	10.0	19.8	24.0	21.7	25.6	21	35
Former	15.8	26.1	15.5	9.6	19.7	6	17
**Alcohol drinking:**							
Never	26.5	22.9	19.8	21.4	18.6	8	10
Occasional	59.8	49.5	60.9	61.9	60.2	10	14
Regular	4.8	9.7	7.9	9.5	6.8	19	19
Stop	8.9	18.0	11.4	7.1	14.4	8	22
**Ever drink driving in past year:**							
Yes	25.4	26.5	42.4	52.9	35.0	20	19
Do not usually drive	8.8	8.2	8.8	9.4	8.3	10	13
**Health and social attributes**							
**Self-assessed health:**							
Poor or very poor	4.6	8.8	3.9	2.4	5.0	7	16
**Chronic conditions:**							
Yes	12.5	29.3	10.8	13.1	9.2	10	13
**Doctor diagnosed depression:**							
Yes	3.4	5.9	6.9	4.8	8.3	13	34
**Social capital:**							
Low trust	38.2	36.9	34.5	35.4	33.9	9	12
Low support	25.5	33.2	20.1	22.6	18.3	9	20
Low interaction	23.3	25.5	28.9	22.6	33.3	9	10
**Injury reported in 2005**							
**Number of injuries:**							
At least once	20.2	29.5	27.9	33.3	24.2	16	16
**Location of injury:**							
Home	5.3	7.1	5.4	5.9	5.0	10	14
Road	5.9	4.8	11.7	16.5	8.3	25	20
Work	3.9	6.4	5.4	9.5	2.5	11	13

For peer review only

Table 3 Injury mortality by International Classification of Diseases (ICD-10), Thai Cohort Study

Types of injury deaths	Number of deaths	Rate per 10000 person-years
**Transport injuries**		
V01-V09 Pedestrian	2	0.2
V20-V29 Motorcycle rider	14	1.6
V40-V49 Car occupant	15	1.7
V50-59 Occupant of pick-up truck or van	3	1.0
V80-V89 Other land transport accident	9	0.1
V89.2 Person injured in unspecified motor vehicle	39	4.5
V90-V94 Water transport	1	0.1
V95-V97 Air and space transport	1	0.1
**Non-transport injuries**		
W00-W19 Falls	2	0.3
W65-W74 Drowning and submersion	10	1.3
W75-W84 Other threats to breathing	1	0.1
W87 Exposure to electric current	2	0.3
X00-X09 Exposure to smoke, fire and flames	2	0.3
X33 Victim of lighting	1	0.1
X38 Victim of flood	1	0.1
X58-X59 Exposure to other unspecified factors	16	2.1
X60-X84 Intentional self-harm		
X65 Intentional self-poisoning	3	0.3
X70 Intentional self-harm by hanging, strangulation and suffocation	7	0.8
X85-Y09 Assault		
X95 Assault by unspecified firearm discharge	22	2.5
X99 Assault by sharp object	3	0.3
Y99 Assault by other unspecified means	5	0.6
Y10-Y34 Event of undetermined intent		
Y18 Poisoning by and exposure to pesticides	1	0.1
Y20 Hanging, strangulation and suffocation	3	0.3
Y22-Y24 Firearm discharge, undetermined intent	13	1.5
Y25 Contact with explosive material	1	0.1
Y28-Y29 Contact with sharp of blunt object	4	0.5
Y34 Unspecified event, undetermined intent	23	2.6

For peer review only

Table 4 Age-sex adjusted and multivariate predictors of injury mortality, Thai Cohort Study

2005 baseline covariates	Multinomial* Adjusted Odds Ratios [95% CI]			
	Age-sex adjusted		Multivariate**	
	Alive	Injury death	Alive	Injury death
**Geo-demographic covariates**				
**Age groups in years:** 20-29		**1.45 [1.06-1.99]**		1.51 [0.99-2.32]
30-39	*Ref*		*Ref*	
40-49		0.76 [0.45-1.28]		0.99 [0.54-1.83]
≥50		1.29 [0.59-2.84]		1.65 [0.61-4.43]
**Sex** *(female)*:	*Ref*		*Ref*	
Male		**3.69 [2.69-5.07]**		**3.87[2.39-6.26]**
**Marital status** *(married)*:	*Ref*		*Ref*	
Not married		1.09 [0.77-1.56]		1.11 [0.73-1.69]
Divorced/widowed		1.55 [0.80-3.02]		1.47 [0.69-3.14]
**Personal monthly income:**				
≤3000Baht		1.24 [0.80-1.93]		1.10 [0.48-1.53]
3001-7000		1.25 [0.89-1.75]		1.15 [0.78-1.69]
7001-20000		0.81 [0.46-1.43]		0.83 [0.43-1.61]
>20000	*Ref*		*Ref*	
**Regions** (*Central/East*):	*Ref*		*Ref*	
Bangkok		0.85 [0.51-1.41]		0.86 [0.48-1.53]
North		1.38 [0.92-2.08]		1.27 [0.80-2.01]
Northeast		1.29 [0.87-1.93]		0.98 [0.61-1.56]
South		**1.96 [1.30-2.97]**		**1.71 [1.05-2.79]**
**Lifecourse residence** *(rural-rural)*:	*Ref*		*Ref*	
Urban-rural		0.91 [0.66-1.27]		0.97 [0.65-1.44]
Rural-urban		1.03 [0.52-2.04]		1.32 [0.73-1.44]
Urban-urban		0.87 [0.59-1.29]		1.12 [0.71-1.77]
**Health and social covariates**				
**Smoking** *(never)*:	*Ref*		*Ref*	
Current		**1.70 [1.07-2.05]**		**1.56 [1.03-2.37]**
Former		0.77 [0.45-1.32]		0.74 [0.47-1.18]
**Alcohol drinking** *(never)*:	*Ref*		*Ref*	
Occasional		0.87 [0.60-1.27]		0.40 [0.12-1.30]
Regular		1.06 [0.58-1.95]		0.38 [0.10-1.40]
Stop		1.08 [0.64-1.84]		0.63 [0.18-2.15]
**Drink driving past year** *(never)*:	*Ref*		*Ref*	
Yes		**1.50 [1.07-2.12]**		**1.49 [1.01-2.20]**
**Self-assessed health** *(good)*:	*Ref*		*Ref*	
Poor or very poor		1.09 [0.58-2.07]		0.75 [0.32-1.71]
**Depression** *(no)*:	*Ref*		*Ref*	
Yes		**2.15 [1.25-3.71]**		**1.91 [1.00-3.69]**
**Chronic illness** *(no)*:	*Ref*		*Ref*	
Yes		0.80 [0.50-1.2]		0.84 [0.50-1.44]
**Social capital:**				
Low social trust (*ref: not low*)		0.84 [0.60-1.19]		0.90 [0.76-1.07]
Low social support (*ref*: not low)		1.29 [0.58-2.84]		1.37 [0.96-1.96]
Low social interaction (*ref*: not low)		0.86 [0.64-1.15]		0.77 [0.50-1.20]
**Injury reported in 2005**				
**Injuries in the past year** *(no)*:	*Ref*		*Ref*	
At least once		**1.41 [1.04-1.92]**		1.12 [0.70-1.82]
**Location of injury:**				
Home (*ref: no*)		1.13 [0.62-2.08]		1.19 [0.56-2.55]
Road (*ref: no*)		**1.81 [1.17-2.79]**		1.58 [0.87-2.84]
Work (*ref: no*)		1.22 [0.66-2.25]		1.15 [0.54-2.44]

*multinomial logistic regression compares the odds of injury deaths to the odds of remaining alive by predictor covariate category values, after adjusting for age-sex or other covariates.

**mutually adjusted for all predictor covariates presented in this table

For peer review only

References

1. Stevenson M. Measuring the global burden of road traffic injury: implications for low-income and middle-income countries. Inj Prev. 2009; 15:1-2.
2. Lim SS, Vos T, Flaxman AD, et al. A comparative risk assessment of burden of disease and injury attributable to 67 risk factors and risk factor clusters in 21 regions, 1990-2010: a systematic analysis for the Global Burden of Disease Study 2010. Lancet. 2012; 380:2224-60.
3. WHO. Global status report on road safety 2013: supporting a decade of action. Geneva: World Health Organization; 2013.
4. WHO. World report on road traffic injury prevention. Geneva: World Health Organization; 2004.
5. Chandran A, Hyder AA, Peek-Asa C. The global burden of unintentional injuries and an agenda for progress. Epidemiol Rev. 2010; 32:110-20.
6. WHO. Global status report on violence prevention. Geneva: World Health Organization; 2014.
7. Bhalla K, Harrison J, Abraham J, et al. Data sources for improving estimates of the global burden of injuries: call for contributors. PLoS Med. 2009; 6 e1 doi: 10.1371/journal.pmed.1000001.
8. Bhalla K, Harrison JE, Fingerhut LA, et al. The global injury mortality data collection of the Global Burden of Disease Injury Expert Group: a publicly accessible research tool. Int J Inj Contr Saf Promot. 2011; 18:249-53.
9. Baker SP, Li G. Epidemiologic approaches to injury and violence. Epidemiol Rev. 2011; 34:1-3.
10. Mathers CD, Fat DM, Inoue M,et al. Counting the dead and what they died from: an assessment of the global status of cause of death data. Bull World Health Organ. 2005; 83:171.
11. Ditsuwan V, Veerman LJ, Barendregt JJ, et al. The national burden of road traffic injuries in Thailand. Popul Health Metr. 2011; 9:2; doi: 10.1186/478-7954-9-2.
12. Chadbunchachai W, Suphanchaimaj W, Settasatien A, et al. Road traffic injuries in Thailand: current situation. J Med Assoc Thai. 2012; 95 Suppl 7:S274-81.
13. Choprapawon C, Porapakkham Y, Sablon O, et al. Thailand's national death registration reform: verifying the causes of death between July 1997 and December 1999. Asia Pac J Public Health. 2005; 17:110-6.
14. Tangcharoensathien V, Faramnuayphol P, Teokul W, et al.A critical assessment of mortality statistics in Thailand: potential for improvements. Bull World Health Organ. 2006; 84:233-8.
15. Rao C, Porapakkham Y, Pattaraarchachai J, et al. Verifying causes of death in Thailand: rationale and methods for empirical investigation. Popul Health Metr. 2010; 8:11.
16. Porapakkham Y, Rao C, Pattaraarchachai J, et al. Estimated causes of death in Thailand, 2005: implications for health policy. Popul Health Metr. 2010; 8:14.
17. Pattaraarchachai J, Rao C, Polprasert W, et al. Cause-specific mortality patterns among hospital deaths in Thailand: validating routine death certification. Popul Health Metr. 2010; 8:12.
18. Yiengprugsawan V, Stephan K, McClure R, et al. Risk factors for injury in a national cohort of 87,134 Thai adults. Public Health. 2012; 126:33-9.
19. Stephan K, Kelly M, McClure R , et al. Distribution of transport injury and related risk behaviours in a large national cohort of Thai adults. Accid Anal Prev. 2011; 43:1062-7.
20. Tawatsupa B, Yiengprugsawan V, Kjellstrom T, et al. Association between heat stress and occupational injury among Thai workers: findings of the Thai Cohort Study. Ind Health. 2013; 51:34-46.
21. Sleigh AC, Seubsman SA, Bain C. Cohort profile: The Thai Cohort of 87,134 Open University students. Int J Epidemiol. 2008; 37:266-72.
22. Seubsman SA, Yiengprugsawan V, Sleigh AC, the Thai Cohort Study T. A large national Thai Cohort Study of the Health-Risk Transition based on Sukhothai Thammathirat Open University students. ASEAN J Open Distance Learn. 2012; 4:58-69.
23. Prasartkul P, Vapattanawong P. The completeness of death registration in Thailand: evidence from demographic surveillance system of the kanchanaburi project. World Health Popul. 2006; 8:43-51.
24. WHO. International Statistical Classification of Diseases and Related Health Problems, 10th Revision. http://apps.who.int/classifications/icd10/browse/2010/en: World Health Organization.; 2011.
25. Leistikow BN, Martin DC, Jacobs J, et al. Smoking as a risk factor for accident death: a meta-analysis of cohort studies. Accid Anal Prev. 2000; 32:397-405.
26. Wen CP, Tsai SP, Cheng TY, et al. Excess injury mortality among smokers: a neglected tobacco hazard. Tobacco control. 2005; 14 Suppl 1:i28-32.
27. Patten SB, Williams JV, Lavorato DH, et al. Major depression and injury risk. Can J Psychiatry. 2010; 55:313-8.

For peer review only

28. Bonura C. Indeterminate geographies of political violence in Southern Thailand. Alternatives. 2008; 33:383-412.
29. McCargo D. Rethinking Thailand's southern violence - Introduction. Crit Asian Stud. 2006; 38:3-10.
30. Taylor B, Irving HM, Kanteres F, et al. The more you drink, the harder you fall: a systematic review and meta-analysis of how acute alcohol consumption and injury or collision risk increase together. Drug Alcohol Depend. 2010; 110:108-16.
31. Connor J, Norton R, Ameratunga S, et al. The contribution of alcohol to serious car crash injuries. Epidemiology. 2004; 15:337-44.
32. Aida J, Kondo K, Hirai H, et al. Assessing the association between all-cause mortality and multiple aspects of individual social capital among the older Japanese. BMC Public Health. 2011; 11:499.
33. Hyyppa MT, Maki J, Impivaara O, et al. Individual-level measures of social capital as predictors of all-cause and cardiovascular mortality: a population-based prospective study of men and women in Finland. Eur J Epidemiol. 2007; 22:589-97.
34. Muennig P, Cohen AK, Palmer A, et al. The relationship between five different measures of structural social capital, medical examination outcomes, and mortality. Soc Sci Med. 2013; 85:18-26.

For peer review only

Predictors of injury mortality: findings from a large national cohort in Thailand

Vasoontara Yiengprugsawan[1§], Janneke Berecki-Gisolf[2], Christopher Bain[3], Roderick McClure[2], Sam-ang Seubsman[1,4], Adrian C Sleigh[1]

[1] National Centre for Epidemiology and Population Health, The Australian National University, Canberra, Australia
[2] Monash Injury Research Institute, Monash University, Melbourne, Australia
[3] Genetics and Population Health Division, Queensland Institute of Medical Research, Brisbane, Queensland, Australia
[4] School of Human Ecology, Sukhothai Thammathirat Open University, Nonthaburi, Thailand

[§] **Correspondence to:** Dr. Vasoontara Yiengprugsawan, The Australian National University, College of Medicine, Biology and Environment, Research School of Population Health, National Centre for Epidemiology and Population Health, Building 62, Mills Rd, Acton 2601, Canberra, Australia Tel: +61 2 6125 2378; Fax: +61 2 6125 0740
Email vasoontara.yieng@anu.edu.au; vasoontara.yieng@gmail.com

For peer review only

Predictors of injury mortality: findings from a large national cohort in Thailand

Abstract

Objective: to present predictors of injury mortality by types of injury and by pre-existing attributes or other individual exposures identified at baseline.

Design: 5-year prospective longitudinal study

Setting: Contemporary Thailand (2005-2010), a country undergoing epidemiological transition

Participants: Data derived from a research cohort of 87,037 distance-learning students enrolled at Sukhothai Thammathirat Open University ~~(STOU)~~ residing nationwide.

Measures: Cohort members completed a comprehensive baseline mail-out questionnaire in 2005 reporting geo-demographic, behavioural, health, and injury data. These responses were matched with national death records using the Thai Citizen ID number. Age-sex adjusted multinomial logistic regression was used to calculate odds ratios linking exposure variables collected at baseline to injury deaths over the next five years.

Results: Statistically significant predictors of injury mortality were being male (Adjusted Odds Ratio 3.87, 95% Confidence Interval 2.39-6.26), residing in the Southern areas (AOR 1.71, 1.05-2.79), being a current smoker (1.56, 1.03-2.37), history of drink driving (AOR 1.49,1.01-2.20) and ever having been diagnosed for depression (AOR 1.91, 1.00-3.69). Other covariates such as being young, having low social support, and reporting road injury in the past year at baseline had moderately predictive AORs ranging from 1.4 to 1.6 but were not statistically significant.

Conclusions: We complemented national death registration with longitudinal data on individual, social, and health attributes. This information is invaluable in yielding insight into certain risk traits such as being a young male, history of drink driving, and history of depression. Such information could be used to inform injury prevention policies and strategies.

For peer review only

Article summary:

Article focus

- Injury is a population health burden in transitional low and middle-income Southeast Asia.
- We investigated a large national cohort of Thai adults for predictors of injury mortality including geo-demographic, social, and health attributes recorded at baseline.

Key messages:

- Injuries constituted almost one third of all deaths in the cohort, and some 40% of those were from transport and nearly 60% were non-transport injuries.
- These injury mortality observations add to our previous Thai work on injury morbidity, highlighting the overall risks, especially depression, male sex, and drink driving.
- The link of the Southern Thai region to non-transport injury deaths most likely reflects the political violence which covers the period since 2004.

Strengths and limitations of this study:

- Injury is a population health burden in transitional low and middle-income Southeast Asia. We investigated a large national cohort of Thai adults for predictors of injury mortality including geo-demographic, social, and health attributes recorded at baseline.
- Injuries constituted almost one third of all deaths in the cohort, and some 40% of those were from transport and nearly 60% were non-transport injuries. These injury mortality observations add to our previous Thai work on injury morbidity, highlighting the overall risks, especially depression, male sex, and drink driving.
- The advantage of our study is its large size, longitudinal design, and comprehensive baseline information. This provides a platform for identification of risks, elimination of confounders, and exploration of causal pathways.
- This study captured the 5-year mortality rate in a generally young adult cohort. Thus there were relatively few deaths. Citizen IDs provided at baseline will enable us to monitor patterns of cohort mortality into the future.

For peer review only

Introduction

Injury remains a major public health challenge worldwide; causing one tenth of global mortality with a heavy burden in developing countries.[1, 2] According to the World Health Organization, at least 1.2 million people are killed from road crashes and an estimated 50 million are injured on roads worldwide each year.[3, 4] Violence and non-transport injuries also accounted for more than 1.3 million deaths and many suffered from serious physical and mental consequences.[5, 6]

Most national injury prevention policies have been introduced in high-income countries. Unfortunately, very few low and middle-income countries have been able to develop such policies due to lack of resources and limited availability of quality injury mortality data.[7, 8] In particular, many developing countries still face the challenges of accurately identifying causes of death from routinely collected national civil registration and vital statistics systems while other sources of data such as police reports and hospital records are never comprehensive and lead to underreporting bias if relied upon as a main source of injury mortality data. Reliable cause-of-death data are important because they enable monitoring of the epidemiological occurrence and public health effects at the population level.[9, 10]

Throughout middle-income Southeast Asia, including Thailand, injury continues to be one of the top ten causes of death.[11, 12] In past decades, Thailand has reformed administrative records to improve the coverage and quality of cause-specific mortality data.[13, 14] Eight years ago, Thailand began to study the ill-defined causes of death by using verbal autopsies and these revealed that besides a high proportion of transport-related deaths, a number of other deaths which were initially recorded as non-specific causes turned out to be suicides, assaults, and drowning.[15-17] These findings shed light on the importance of non-transport injuries in addition to the burden of transport injuries.

This study is based on a large national cohort in Thailand which has been followed to investigate health-risk transitions of Thai adults since 2005. The cohort database includes comprehensive information on individual characteristics, social demography, health behaviours and specific diseases, as well as history of injuries. Our previous research based on this cohort has examined risk factors associated with injury morbidity.[18-20] Now successful mortality data linkage

For peer review only

through the Thai Ministry of Interior and Ministry of Public Health allows us to analyse injury-related deaths among the cohort over the first five years (2005-10).

Informed by our earlier research on injury morbidity, and by related published information, this study has investigated injury in more depth using mortality as the outcome. Our study linked cohort outcomes (survival, non-injury death, injury death) to an array of relevant exposures recorded at baseline including geo-demographic attributes, social covariates, health and psychological states, and health-risk behaviours. This analysis is both prospective and cohort-based and fills an important gap regarding our knowledge of injury risks as an emerging public health problem in a middle-income Asian country going through the health-risk transition.

Methods

Study population and data collection

This analysis is part of the overarching Thai Cohort Study (TCS), an ongoing epidemiological investigation of changing patterns of health risks and outcomes. Data derive from a research cohort of 87,037 distance-learning adult students enrolled at Sukhothai Thammathirat Open University ~~(STOU)~~, who resided all over Thailand and completed the baseline comprehensive mail-out health questionnaire in 2005 (response rate 44%). The cohort participants recapitulated well the distance learning student body at STOU and share certain geo-demographic attributes with the general Thai population (mean age was 29 years in 2005, slightly more than half were females, half resided in urban areas).[21, 22] The baseline questionnaire gathered data on a wide range of topics including age, sex, income, marital status, health status, doctor-diagnosed diseases, health-risk behaviors including smoking and drinking, social capital, and history of injury.

Mortality data

The completeness of death registration in Thailand was 86% from 1950-2000[10], but over the last decade coverage improved to 95%.[23] A powerful feature of our study is that all cohort members have provided their Thai Citizen ID number enabling detection and analysis of deaths into the future. These confidential ID numbers were safeguarded and stored at STOU in a secure office on the main campus with 24-hour guards on patrol. The working files of these data were de-identified and no individual information will be released or displayed in any format. To detect

For peer review only

deaths, the Bangkok TCS team periodically matched the cohort against national death records from the Ministry of Interior using the Citizen ID number. At a later stage the Thai Ministry of Public Health expanded these death records by adding the standard International Classification of Diseases (ICD-10)[24] to identify causes of death.

Up until March 2010, there were a total of 580 deaths among the Thai Cohort Study participants. According to the ICD-10 codes, there were 376 deaths from non-injury causes including ill-defined causes of death. For the purpose of this study, these will not be broken down and will be designated as 'other deaths'. For our injury-focused death analysis, there were 204 deaths from external causes, including 84 deaths from transport accidents. Among the 120 non-transport injury deaths, there were 35 deaths from miscellaneous external causes, 10 deaths from intentional self-harm, 30 deaths from assault, and 45 deaths from 'unspecified events of undetermined intent'.

Exposures and confounders

In our analysis, exposures of interest and potential confounders from the 2005 baseline questionnaire included the following geo-demographic variables: age (4 categories), sex, marital status (married, not married, divorced/ widowed), personal monthly income (≤3000 Baht, 3001-7000, 70~~0~~01-10000, 10001-20000, >20000), regions (Central/East, Bangkok, North, Northeast, South), and lifecourse urbanisation (residence at age 12 years old and at baseline: rural-rural, rural-urban, urban-rural, urban-urban). As well, a history of injury in the past year was reported at 2005 baseline, including the frequency and location of injuries reported.

Also analysed as exposures of interest were certain social covariates, several health states, and important health-risk behaviours. These behaviours included smoking and alcohol drinking which have been shown to be independently associated with injury.[25, 26] Smoking status includes never, current, and former and alcohol status includes never, occasional, regular, and former. In addition, at baseline cohort members were asked "in the last year have you driven a motor vehicle after consuming 3 or more glasses of alcohol" (ie drink driving). Other health-related attributes included self-assessed health and chronic metabolic or cardiovascular disorders (e.g. diabetes, hypertension). A history of doctor-diagnosed depression has previously been shown to be an injury risk[18, 27] and is also included in the model. Social capital was dichotomoised for analyses (low or not low) in three domains: trust (whether people can be trusted), support (from family, friends, colleagues), and interaction (with family, friends, neighbours).

For peer review only

Data processing and statistical analysis

Questionnaire responses were digitised by optical scanning and subsequently edited using Thai Scandevet, SQL and SPSS software. For analysis we used Stata version 12. Individuals with missing data for given analyses were excluded (<5%), so totals vary slightly according to available information. We described the distribution of deaths by demographic, social, and health covariates, by cause of death, and by types of injury (transport and non-transport injuries). In addition, we presented death rates per 10,000 person-years of exposure. We then used age-sex adjusted multinomial logistic regression linking the above covariates to three possible outcomes: alive (reference), deaths from other causes, and deaths from injury (study outcome). The final analytical model mutually adjusted for all covariates and reported Adjusted Odds Ratios (AOR) and 95% Confidence Intervals.

Results

Among Thai cohort members at baseline in 2005 (Table 1), about one third of the cohort were less than 30 years old, slightly more than half were female, about 40% reported monthly income of less than 7000 Baht per month (175 US$ in 2005), 18% lived in Bangkok, and about half were urban residents. Injury deaths were more likely to affect males (73.7%, 15 vs 23 per 10000 person-years for transport vs non-transport injuries). Also notable, injury deaths were disproportionately frequent in the Southern region (20.6%, 12 vs 25 per 10000 person-years for transport vs non-transport injuries).

For social and health attributes (Table 2), a history of ever drink driving in the past year was more common among injury deaths (42.4% compared to 26.5% for other deaths or 25.4% for alive) and notably higher for transport than non-transport injuries (52.9% vs 35.0%). Cohort members who died from non-injury related causes were twice as likely to have reported poor self-assessed health at baseline and three times as likely to have reported metabolic and cardiovascular chronic conditions. Cohort members who died from injury reported higher rates of ever having been doctor-diagnosed depression (6.9% compared to 3.4% among non-deaths). As well, a history of depression was much more frequent for those who died from non-transport injuries than for transport injuries (34 vs 13 per 10000 person-years). At baseline in 2005, about 20% of cohort members overall reported injury at least once in the past year compared to 33.3% of cohort participants who died from transport injury.

For peer review only

In addition to analysing by injury types, we also tabulated the death rates according to the International Classification of Diseases (Table 3). Within transport injury mortality, rates per 10000 person-years for motorcycle riders and car occupants were 1.6 and 1.7, respectively. There were also 4.5 per 10000 person-years who died in unspecified motor vehicles. Among non-transport injury deaths, the rate per 10000 person-years of assault by firearm discharge was 2.5 with an additional 1.5 deaths from firearm discharge with undetermined intent. Deaths from drowning and submersion were 1.3 per 10000 person-years. Intentional self-harm deaths included self-poisoning and hanging-strangulation-suffocation with death rates of 0.3 and 0.8 per 10000 person-years, respectively.

To examine predictors of injury deaths (Table 4), we used multinomial logistic regression with three outcome categories: alive (reference), non-injury deaths, and injury deaths (study outcome). Highlighted in bold were results that were statistically significant at $p<0.05$. In the first column of odds ratios, the results are adjusted for age and sex; in the second column the odds ratios are adjusted for all covariates. All odds ratios compare the odds of injury death to the odds of staying alive.

We first calculate age-sex adjusted odds ratios for each potential exposure and for covariates. In the age-sex adjusted mode, being younger than 30 years, being male, residing in the South, currently smoking, drink driving in the past year, ever having been diagnosed for depression~~, having low social support and~~, injury incidence in the year preceding baseline, and reported road injury in 2005, all were associated with injury mortality.

We then proceed to multivariate analysis (Table 4). After mutually adjusting for all tabulated covariates, statistically significant predictors of injury mortality were being male (Adjusted Odds Ratio, AOR 3.87), residing in the Southern areas (AOR 1.71), being a current smoker (AOR 1.56), history of drink driving (AOR 1.49) and ever having been diagnosed for depression (AOR 1.91). Other covariates such as being young, having low social support, and reporting road injury in the past year at baseline had predictive AORs ranging from 1.4 to 1.6, but these substantive estimates were not statistically significant for overall injury. Further investigation into types of injury (data not shown) revealed that, younger age was a strong predictor for transport injury deaths (AOR 4.12, 95% 1.03-16.5) and low social support for non-transport injury deaths (AOR 1.64, 95% 1.04-2.59).

For peer review only

In marked contrast to the injury deaths, cohort members who died from other causes had different sets of statistically significant risks at 2005 baseline which included older age, residing in Bangkok, reporting poor self-assessed health, and having metabolic or cardiovascular chronic conditions (data not shown). The only risk factor that non-injury deaths have in common with injury deaths was being a current smoker.

Discussion

This study is embedded in an overarching investigation of health-risk transition in Thailand, where similar to middle-income Southeast Asian countries; injury has been and still is a major population health burden. We make use of our large cohort of adults to investigate risk factors associated with injury mortality, linking deaths over five years (2005-2010) to individual characteristics and social and health attributes recorded at baseline (2005). The results revealed that injuries constituted almost one third of all deaths in the cohort, and some 40% of those were from transport and nearly 60% were non-transport injuries. Connecting to baseline information provided five years earlier, we identified epidemiologically and statistically significant predictors of injury mortality. These predictors included certain geo-demographic characteristics (male sex, Southern residents), health risk behaviours (smoking and history of drink driving), and adverse health diagnoses (depression).

Our injury mortality findings provide further information adding to our previous knowledge on risk factors associated with injury morbidity in the cohort, especially being male, having a history of depression, and drink driving.[18-20] The effect of smoking on injury was also shown in other studies after controlling for covariates including alcohol drinking. Plausible explanations include accidental fire hazard and distraction or inattention for road traffic hazards.[25, 26] Non-transport injury deaths presented here reflected the current political situation with violence in the Southern part of Thailand since 2004.[28, 29] For covariates with substantial (but not statistically significant) point estimates of overall injury death (young age, low social support, history of road injury), AORs ranged from 1.4-1.6.

Our results support other published international information on risk factors related to injury mortality, showing young males and drunk drivers at high risk of road crash death.[30, 31] A systematic review on alcohol consumption and collision risk concluded that there is no safe

level of drinking and even less than two drinks per occasion can almost double the odds of most types of injury.[30]

Our study also found social capital to be protective against injury mortality, supporting previous research.[32-34] Indeed, we found low social support to be a predictor of non-transport injury mortality. But social support did not relate to transport injury mortality. Despite the relatively small number of deaths in our study, we found that low social support was particularly associated with intentional self-harm and assault but these associations were not statistically significant.

The advantage of our study is its large participation by Thai adults who completed a comprehensive 20-page baseline questionnaire in 2005, reporting on a wide array of social and health characteristics. This provided a platform for investigation of causal pathways and elimination of many confounders. While our cohort members share similar distribution of sex, modest income, and geographical residence with the general Thai population. However, all cohort members completed high school education which facilitated their ability to respond to our detailed questionnaire. We also noted that a few (<5%) cohort members were 'missing' data for variables used in various models but this problem was not numerically significant. We acknowledge that our study captured the 5-year mortality rate after the baseline questionnaire in 2005 and the number of deaths was relatively small. However, the citizen IDs provided by cohort members at the baseline can be used to monitor patterns of mortality into the future and eventually a full account of cohort mortality will be possible.

Our research contributes to limited longitudinal evidence linking risk factors to injury death and is one of the first studies in middle-income Southeast Asia. We have achieved our aim of identifying vulnerable population subgroups at risk of injury deaths. As well, we complemented routinely collected administration death registration with a longitudinal health assessment providing information on individual, social, and health history factors. This information is invaluable in yielding insight into certain risk traits such as being a young male, reporting and having a past medical diagnosis of depression, which could inform injury prevention policies and strategies suitable for transitional countries with limited resources and changing patterns of mortality.

For peer review only

Contributors

VY conceptualized, analysed and drafted the manuscript. JB-G, RM, CB provided inputs to improve the draft. SS and AS designed and executed the Thai Cohort Study. AS provided comments and guidance on ~~various~~ revisions. All authors read and approved the final manuscript.

Author footnote

The Thai Cohort Study Team

Thailand: Jaruwan Chokhanapitak, Suttanit Hounthasarn, Suwanee Khamman, Daoruang Pandee, Suttinan Pangsap, Tippawan Prapamontol, Janya Puengson, Sam-ang Seubsman, Boonchai Somboonsook, Nintita Sripaiboonkij, Pathumvadee Somsamai, Duangkae Vilainerun, Wanee Wimonwattanaphan, Tewarit Somkotra, Benjawan Tawatsupa, Wimalin Rimpeekool.

Australia: Chris Bain, Emily Banks, Cathy Banwell, Bruce Caldwell, Gordon Carmichael, Tarie Dellora, Jane Dixon, Sharon Friel, David Harley, Matthew Kelly, Tord Kjellstrom, Lynette Lim, Anthony McMichael, Tanya Mark, Penny Haora, Adrian Sleigh, Lyndall Strazdins, Susan Jordan, Roderick McClure, Janneke Berecki-Gisolf, Vasoontara Yiengprugsawan.

Acknowledgments

We thank the staff at Sukhothai Thammathirat Open University (STOU) who assisted with student contact and the STOU students who are participating in the cohort study. We also thank Dr Bandit Thinkamrop and his team from Khon Kaen University for support on initial data processing. As well we would like thank the Thai Ministry of Interior and Ministry of Public Health for mortality investigation and cause of death data linkage. Matthew Kelly and Peter Sbirakos provided language editorial support.

Funding

This study was supported by the International Collaborative Research Grants Scheme with joint grants from the Wellcome Trust UK (GR071587MA) and the Australian National Health and Medical Research Council (268055), and as a global health grant from the NHMRC (585426).

Ethical approval

Informed written consent was obtained from all participants. All students were advised that they could withdraw, or not participate, without any effect on their academic progress. The questionnaires never sought sensitive personal information and no biological samples were taken. Ethics approval was obtained from Sukhothai Thammathirat Open University Research and Development Institute (protocol 0522/10) and the Australian National University Human Research Ethics Committee (protocols 2004/344 and 2009/570).

Competing interests

None declared.

Data sharing statement

Data are available through a data access agreement which includes guarantees regarding ethical conduct and scientific quality of any proposed analyses and publications. Anyone wanting access should contact Professor Adrian Sleigh or Associate Professor Sam-ang Seubsman (Thai Cohort Study Principal Investigators).

For peer review only

Table 1 Distribution of mortality by baseline social and geo-demographic attributes, Thai Cohort Study

Cohort attributes	Vital status by attributes %					Incidence per 10000 person-years	
	Alive (86457)	Other deaths (376)	Injury deaths (204)	Injury deaths (204)		Transport (84)	Non-transport (120)
				Transport (84)	Non-transport (120)		
**Age groups in years:**							
18-29	31.4	27.7	27.9	20.2	25.7	6	15
30-39	53.6	29.5	59.3	69.1	51.4	12	14
40-49	12.5	25.8	9.3	6.0	17.1	5	13
≥50	2.4	17.0	3.4	4.8	5.7	19	14
**Sex:**							
Males	45.2	65.6	73.7	69.4	76.7	15	23
**Marital status:**							
Married	38.8	50.1	35.0	30.9	37.7	8	14
Not married	56.8	41.9	60.1	64.3	57.1	11	14
Divorced/widowed	4.4	8.0	4.9	4.8	5.0	11	16
**Personal monthly income:**							
≤3000 Baht	11.0	14.9	14.6	11.9	16.5	10	20
3001-7000	30.9	22.4	33.2	39.3	28.7	13	13
7001-10000	23.3	16.6	23.6	26.2	21.7	11	13
10001-20000	24.2	28.7	20.6	16.7	23.5	7	13
>20000	10.5	17.4	8.0	6.0	9.6	6	12
**Regions:**							
Central/East	30.7	30.7	24.9	23.8	24.2	8	11
Bangkok	17.2	18.7	10.7	9.4	11.7	5	9
North	18.2	22.7	21.0	23.5	19.2	13	15
Northeast	20.9	21.6	23.4	25.9	21.7	12	14
South	13.0	12.3	20.6	16.7	23.3	12	25
**Lifecourse residence:**							
Rural-rural	43.3	42.3	46.6	38.1	52.5	8	17
Rural-urban	31.5	25.0	16.7	28.6	30.8	9	13
Urban-rural	4.2	6.9	4.4	7.1	2.5	16	8
Urban-urban	19.7	22.3	16.7	22.6	12.5	11	9

For peer review only

Table 2 Mortality by baseline health-risk behaviours and states, social attributes, and history of injury, Thai Cohort Study

Social and health attributes	Vital status by attributes %					Incidence per 10000 person-years	
	Alive (86457)	Other deaths (376)	Injury deaths (204)	Injury deaths (204)		Transport (84)	Non-transport (120)
				Transport (84)	Non-transport (120)		
**Health-risk attributes**							
**Smoking:**							
Never	72.3	51.1	57.5	66.3	51.3	9	10
Current	10.0	19.8	24.0	21.7	25.6	21	35
Former	15.8	26.1	15.5	9.6	19.7	6	17
**Alcohol drinking:**							
Never	26.5	22.9	19.8	21.4	18.6	8	10
Occasional	59.8	49.5	60.9	61.9	60.2	10	14
Regular	4.8	9.7	7.9	9.5	6.8	19	19
Stop	8.9	18.0	11.4	7.1	14.4	8	22
**Ever drink driving in past year:**							
Yes	25.4	26.5	42.4	52.9	35.0	20	19
Do not usually drive	8.8	8.2	8.8	9.4	8.3	10	13
**Health and social attributes**							
**Self-assessed health:**							
Poor or very poor	4.6	8.8	3.9	2.4	5.0	7	16
**Chronic conditions:**							
Yes	12.5	29.3	10.8	13.1	9.2	10	13
**Doctor diagnosed depression:**							
Yes	3.4	5.9	6.9	4.8	8.3	13	34
**Social capital:**							
Low trust	38.2	36.9	34.5	35.4	33.9	9	12
Low support	25.5	33.2	20.1	22.6	18.3	9	20
Low interaction	23.3	25.5	28.9	22.6	33.3	9	10
**Injury reported in 2005**							
**Number of injuries:**							
At least once	20.2	29.5	27.9	33.3	24.2	16	16
**Location of injury:**							
Home	5.3	7.1	5.4	5.9	5.0	10	14
Road	5.9	4.8	11.7	16.5	8.3	25	20
Work	3.9	6.4	5.4	9.5	2.5	11	13

For peer review only

Table 3 Injury mortality by International Classification of Diseases (ICD-10), Thai Cohort Study

Types of injury deaths	Number of deaths	Rate per 10000 person-years
**Transport injuries**		
V01-V09 Pedestrian	2	0.2
V20-V29 Motorcycle rider	14	1.6
V40-V49 Car occupant	15	1.7
V50-59 Occupant of pick-up truck or van	3	1.0
V80-V89 Other land transport accident	9	0.1
V89.2 Person injured in unspecified motor vehicle	39	4.5
V90-V94 Water transport	1	0.1
V95-V97 Air and space transport	1	0.1
**Non-transport injuries**		
W00-W19 Falls	2	0.3
W65-W74 Drowning and submersion	10	1.3
W75-W84 Other threats to breathing	1	0.1
W87 Exposure to electric current	2	0.3
X00-X09 Exposure to smoke, fire and flames	2	0.3
X33 Victim of lighting	1	0.1
X38 Victim of flood	1	0.1
X58-X59 Exposure to other unspecified factors	16	2.1
X60-X84 Intentional self-harm		
X65 Intentional self-poisoning	3	0.3
X70 Intentional self-harm by hanging, strangulation and suffocation	7	0.8
X85-Y09 Assault		
X95 Assault by unspecified firearm discharge	22	2.5
X99 Assault by sharp object	3	0.3
Y99 Assault by other unspecified means	5	0.6
Y10-Y34 Event of undetermined intent		
Y18 Poisoning by and exposure to pesticides	1	0.1
Y20 Hanging, strangulation and suffocation	3	0.3
Y22-Y24 Firearm discharge, undetermined intent	13	1.5
Y25 Contact with explosive material	1	0.1
Y28-Y29 Contact with sharp of blunt object	4	0.5
Y34 Unspecified event, undetermined intent	23	2.6

For peer review only

Table 4 Age-sex adjusted and multivariate predictors of injury mortality, Thai Cohort Study

2005 baseline covariates	Multinomial* Adjusted Odds Ratios [95% CI]			
	Age-sex adjusted		Multivariate**	
	Alive	Injury death	Alive	Injury death
**Geo-demographic covariates**				
**Age groups in years:** 20-29		**1.45 [1.06-1.99]**		1.51 [0.99-2.32]
30-39	*Ref*		*Ref*	
40-49		0.76 [0.45-1.28]		0.99 [0.54-1.83]
≥50		1.29 [0.59-2.84]		1.65 [0.61-4.43]
**Sex** *(female):*	*Ref*		*Ref*	
Male		**3.69 [2.69-5.07]**		**3.87[2.39-6.26]**
**Marital status** *(married):*	*Ref*		*Ref*	
Not married		1.09 [0.77-1.56]		1.11 [0.73-1.69]
Divorced/widowed		1.55 [0.80-3.02]		1.47 [0.69-3.14]
**Personal monthly income:**				
≤3000Baht		1.24 [0.80-1.93]		1.10 [0.48-1.53]
3001-7000		1.25 [0.89-1.75]		1.15 [0.78-1.69]
7001-20000		0.81 [0.46-1.43]		0.83 [0.43-1.61]
>20000	*Ref*		*Ref*	
**Regions** (*Central/East*):	*Ref*		*Ref*	
Bangkok		0.85 [0.51-1.41]		0.86 [0.48-1.53]
North		1.38 [0.92-2.08]		1.27 [0.80-2.01]
Northeast		1.29 [0.87-1.93]		0.98 [0.61-1.56]
South		**1.96 [1.30-2.97]**		**1.71 [1.05-2.79]**
**Lifecourse residence** *(rural-rural):*	*Ref*		*Ref*	
Urban-rural		0.91 [0.66-1.27]		0.97 [0.65-1.44]
Rural-urban		1.03 [0.52-2.04]		1.32 [0.73-1.44]
Urban-urban		0.87 [0.59-1.29]		1.12 [0.71-1.77]
**Health and social covariates**				
**Smoking** *(never):*	*Ref*		*Ref*	
Current		**1.70 [1.07-2.05]**		**1.56 [1.03-2.37]**
Former		0.77 [0.45-1.32]		0.74 [0.47-1.18]
**Alcohol drinking** *(never):*	*Ref*		*Ref*	
Occasional		0.87 [0.60-1.27]		0.40 [0.12-1.30]
Regular		1.06 [0.58-1.95]		0.38 [0.10-1.40]
Stop		1.08 [0.64-1.84]		0.63 [0.18-2.15]
**Drink driving past year** *(never):*	*Ref*		*Ref*	
Yes		**1.50 [1.07-2.12]**		**1.49 [1.01-2.20]**
**Self-assessed health** *(good):*	*Ref*		*Ref*	
Poor or very poor		1.09 [0.58-2.07]		0.75 [0.32-1.71]
**Depression** *(no):*	*Ref*		*Ref*	
Yes		**2.15 [1.25-3.71]**		**1.91 [1.00-3.69]**
**Chronic illness** *(no):*	*Ref*		*Ref*	
Yes		0.80 [0.50-1.2]		0.84 [0.50-1.44]
**Social capital:**				
Low social trust (*ref: not low*)		0.84 [0.60-1.19]		0.90 [0.76-1.07]
Low social support (*ref*: not low)		1.29 [0.58-2.84]		1.37 [0.96-1.96]
Low social interaction (*ref*: not low)		0.86 [0.64-1.15]		0.77 [0.50-1.20]
**Injury reported in 2005**				
**Injuries in the past year** *(no):*	*Ref*		*Ref*	
At least once		**1.41 [1.04-1.92]**		1.12 [0.70-1.82]
**Location of injury:**				
Home (*ref: no*)		1.13 [0.62-2.08]		1.19 [0.56-2.55]
Road (*ref: no*)		**1.81 [1.17-2.79]**		1.58 [0.87-2.84]
Work (*ref: no*)		1.22 [0.66-2.25]		1.15 [0.54-2.44]

*multinomial logistic regression compares the odds of injury deaths to the odds of remaining alive by predictor covariate category values, after adjusting for age-sex or other covariates.

**mutually adjusted for all predictor covariates presented in this table

For peer review only

References

1. Stevenson M. Measuring the global burden of road traffic injury: implications for low-income and middle-income countries. Inj Prev. 2009; 15:1-2.
2. Lim SS, Vos T, Flaxman AD, Danaei G, Shibuya K, Adair-Rohani H, et al. A comparative risk assessment of burden of disease and injury attributable to 67 risk factors and risk factor clusters in 21 regions, 1990-2010: a systematic analysis for the Global Burden of Disease Study 2010. Lancet. 2012; 380:2224-60.
3. WHO. Global status report on road safety 2013: supporting a decade of action. Geneva: World Health Organization; 2013.
4. WHO. World report on road traffic injury prevention. Geneva: World Health Organization; 2004.
5. Chandran A, Hyder AA, Peek-Asa C. The global burden of unintentional injuries and an agenda for progress. Epidemiol Rev. 2010; 32:110-20.
6. WHO. Global status report on violence prevention. Geneva: World Health Organization; 2014.
7. Bhalla K, Harrison J, Abraham J, Borse NN, Lyons R, Boufous S, et al. Data sources for improving estimates of the global burden of injuries: call for contributors. PLoS Med. 2009; 6 e1 doi: 10.1371/journal.pmed.1000001.
8. Bhalla K, Harrison JE, Fingerhut LA, Shahraz S, Abraham J, Yeh PH, et al. The global injury mortality data collection of the Global Burden of Disease Injury Expert Group: a publicly accessible research tool. Int J Inj Contr Saf Promot. 2011; 18:249-53.
9. Baker SP, Li G. Epidemiologic approaches to injury and violence. Epidemiol Rev. 2011; 34:1-3.
10. Mathers CD, Fat DM, Inoue M, Rao C, Lopez AD. Counting the dead and what they died from: an assessment of the global status of cause of death data. Bull World Health Organ. 2005; 83:171.
11. Ditsuwan V, Veerman LJ, Barendregt JJ, Bertram M, Vos T. The national burden of road traffic injuries in Thailand. Popul Health Metr. 2011; 9:2; doi: 10.1186/478-7954-9-2.
12. Chadbunchachai W, Suphanchaimaj W, Settasatien A, Jinwong T. Road traffic injuries in Thailand: current situation. J Med Assoc Thai. 2012; 95 Suppl 7:S274-81.
13. Choprapawon C, Porapakkham Y, Sablon O, Panjajaru R, Jhantharatat B. Thailand's national death registration reform: verifying the causes of death between July 1997 and December 1999. Asia Pac J Public Health. 2005; 17:110-6.
14. Tangcharoensathien V, Faramnuayphol P, Teokul W, Bundhamcharoen K, Wibulpholprasert S. A critical assessment of mortality statistics in Thailand: potential for improvements. Bull World Health Organ. 2006; 84:233-8.
15. Rao C, Porapakkham Y, Pattaraarchachai J, Polprasert W, Swampunyalert N, Lopez AD. Verifying causes of death in Thailand: rationale and methods for empirical investigation. Popul Health Metr. 2010; 8:11.
16. Porapakkham Y, Rao C, Pattaraarchachai J, Polprasert W, Vos T, Adair T, et al. Estimated causes of death in Thailand, 2005: implications for health policy. Popul Health Metr. 2010; 8:14.
17. Pattaraarchachai J, Rao C, Polprasert W, Porapakkham Y, Pao-In W, Singwerathum N, et al. Cause-specific mortality patterns among hospital deaths in Thailand: validating routine death certification. Popul Health Metr. 2010; 8:12.
18. Yiengprugsawan V, Stephan K, McClure R, Kelly M, Seubsman S, Bain C, et al. Risk factors for injury in a national cohort of 87,134 Thai adults. Public Health. 2012; 126:33-9.
19. Stephan K, Kelly M, McClure R, Seubsman SA, Yiengprugsawan V, Bain C, et al. Distribution of transport injury and related risk behaviours in a large national cohort of Thai adults. Accid Anal Prev. 2011; 43:1062-7.
20. Tawatsupa B, Yiengprugsawan V, Kjellstrom T, Berecki-Gisolf J, Seubsman SA, Sleigh A. Association between heat stress and occupational injury among Thai workers: findings of the Thai Cohort Study. Ind Health. 2013; 51:34-46.
21. Sleigh AC, Seubsman SA, Bain C. Cohort profile: The Thai Cohort of 87,134 Open University students. Int J Epidemiol. 2008; 37:266-72.
22. Seubsman SA, Yiengprugsawan V, Sleigh AC, the Thai Cohort Study T. A large national Thai Cohort Study of the Health-Risk Transition based on Sukhothai Thammathirat Open University students. ASEAN J Open Distance Learn. 2012; 4:58-69.
23. Prasartkul P, Vapattanawong P. The completeness of death registration in Thailand: evidence from demographic surveillance system of the kanchanaburi project. World Health Popul. 2006; 8:43-51.
24. WHO. International Statistical Classification of Diseases and Related Health Problems, 10th Revision. http://apps.who.int/classifications/icd10/browse/2010/en: World Health Organization.; 2011.
25. Leistikow BN, Martin DC, Jacobs J, Rocke DM, Noderer K. Smoking as a risk factor for accident death: a meta-analysis of cohort studies. Accid Anal Prev. 2000; 32:397-405.

For peer review only

26. Wen CP, Tsai SP, Cheng TY, Chan HT, Chung WS, Chen CJ. Excess injury mortality among smokers: a neglected tobacco hazard. Tobacco control. 2005; 14 Suppl 1:i28-32.
27. Patten SB, Williams JV, Lavorato DH, Eliasziw M. Major depression and injury risk. Can J Psychiatry. 2010; 55:313-8.
28. Bonura C. Indeterminate geographies of political violence in Southern Thailand. Alternatives. 2008; 33:383-412.
29. McCargo D. Rethinking Thailand's southern violence - Introduction. Crit Asian Stud. 2006; 38:3-10.
30. Taylor B, Irving HM, Kanteres F, Room R, Borges G, Cherpitel C, et al. The more you drink, the harder you fall: a systematic review and meta-analysis of how acute alcohol consumption and injury or collision risk increase together. Drug Alcohol Depend. 2010; 110:108-16.
31. Connor J, Norton R, Ameratunga S, Jackson R. The contribution of alcohol to serious car crash injuries. Epidemiology. 2004; 15:337-44.
32. Aida J, Kondo K, Hirai H, Subramanian SV, Murata C, Kondo N, et al. Assessing the association between all-cause mortality and multiple aspects of individual social capital among the older Japanese. BMC Public Health. 2011; 11:499.
33. Hyyppa MT, Maki J, Impivaara O, Aromaa A. Individual-level measures of social capital as predictors of all-cause and cardiovascular mortality: a population-based prospective study of men and women in Finland. Eur J Epidemiol. 2007; 22:589-97.
34. Muennig P, Cohen AK, Palmer A, Zhu W. The relationship between five different measures of structural social capital, medical examination outcomes, and mortality. Soc Sci Med. 2013; 85:18-26.

For peer review only
